# Aggregation induced emission based fluorescent and colorimetric sensor for selective detection of Fe^2+^: experimental and computational insights with real-time applications

**DOI:** 10.1039/d6ra06979g

**Published:** 2026-11-09

**Authors:** Bushra Jadoon, Alam Shabbir, Asad Muhammad Khan, Mohammed A. Assiri, Umar Farooq, Sohail Anjum Shahzad

**Affiliations:** a Department of Chemistry, COMSATS University Islamabad, Abbottabad Campus University Road Abbottabad 22060 Pakistan umarf@cuiatd.edu.pk sashahzad@cuiatd.edu.pk; b Department of Chemistry, Faculty of Science, King Khalid University P.O. Box 9004 Abha 61413 Saudi Arabia; c Central Labs, King Khalid University P.O. Box 960, AlQura'a Abha 61413 Saudi Arabia

## Abstract

A highly responsive fluorescent and colorimetric sensor (*N*^2^,*N*^6^-bis(4-(phenylamino)phenyl)pyridine-2,6-dicarboxamide) DPD has been developed for the selective detection of Fe^2+^ ions. DPD was readily synthesized through a nucleophilic substitution reaction and characterized through spectroscopic techniques. Sensor DPD displayed aggregation-induced emission (AIE) accompanied by an increase in fluorescence intensity with a bathochromic shift. The aggregation behavior of DPD was further examined by dynamic light scattering analysis (DLS). Among the different metal ions investigated, DPD showed a distinct and highly selective response toward Fe^2+^, resulting in substantial fluorescence quenching due to photo-induced electron transfer (PET) and the paramagnetic behaviour of Fe^2+^. The interaction of DPD with Fe^2+^ was investigated by fluorescence and UV/visible spectroscopy, FTIR spectroscopy, mass spectrometry, and ^1^H NMR titration and cyclic voltammetry which collectively provide the evidence of DPD@Fe^2+^ interaction. Theoretical calculations, including frontier molecular orbital analysis and QTAIM analysis, were performed to support the experimental findings. Sensor DPD was successfully employed for the colorimetric detection of Fe^2+^ in the solid phase and in real potato samples. Furthermore, the sensor was evaluated for the detection of acidic vapors.

## Abstract 2

A highly responsive fluorescent and colorimetric sensor (*N*^2^,*N*^6^-bis(4-(phenylamino)phenyl)pyridine-2,6-dicarboxamide) DPD has been developed for the selective detection of Fe^2+^ ions.

## Graphical abstract

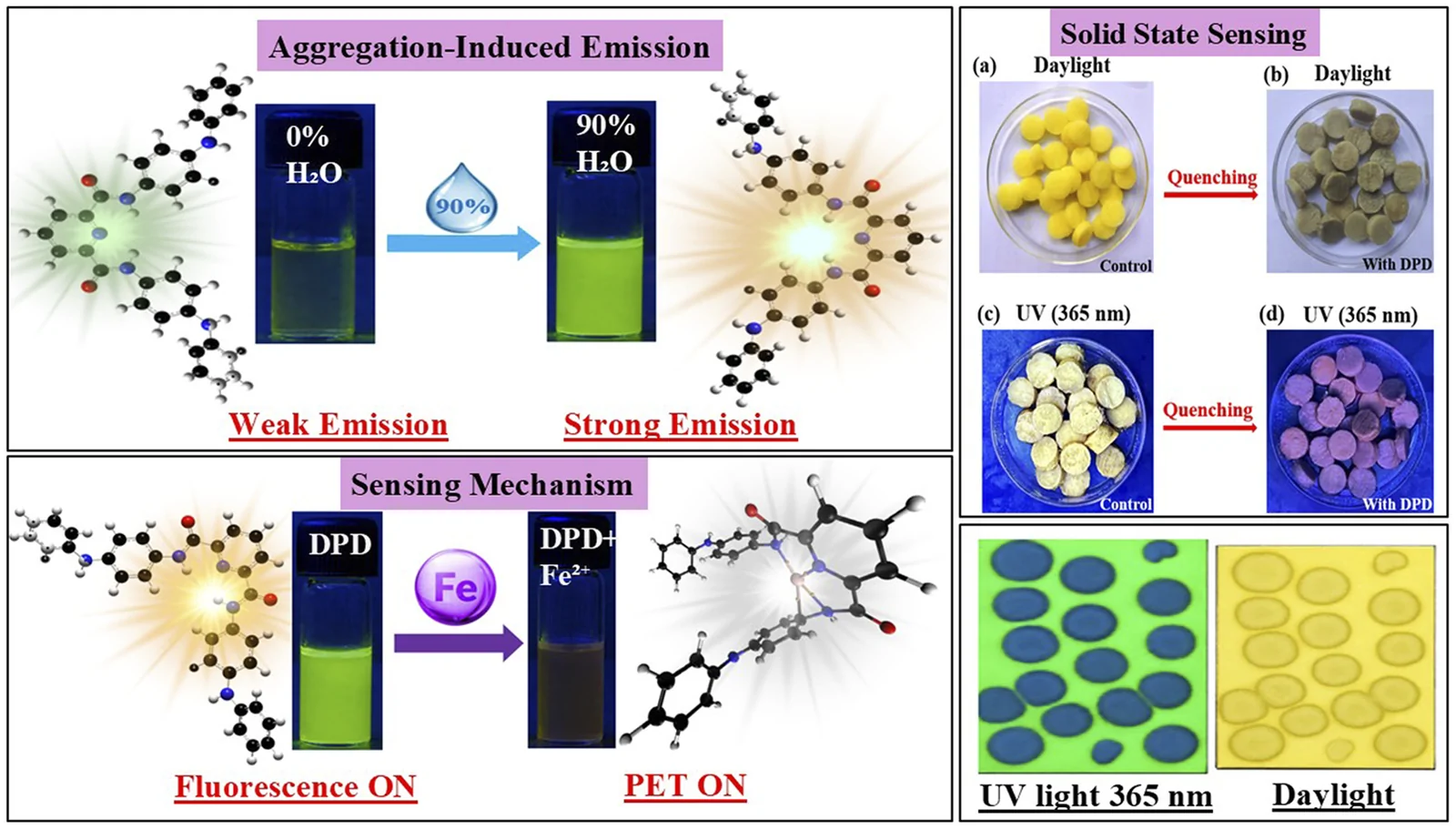

## Introduction

Iron is a redox-active metal ion that is fundamental to human physiology, making it one of the most essential and abundant dietary micronutrients.^1^ On an individual basis, iron is the most common transition metal, with normal body concentrations of about 45 mg kg^−1^ in men and 35 mg kg^−1^ in women. Haemoglobin in erythrocytes, which make up more than 60% of the body's iron, uses iron as the essential binding site for oxygen, sustaining cellular respiration.^2^ The majority of the remaining iron is either stored in the ferritin protein or is an essential cofactor in the active sites of important enzymes that are involved in redox reactions.^3^ This includes enzymes involved in a wide variety of metabolic processes such as DNA synthesis (ribonucleotide reductase), electron transport (mitochondrial complexes, cytochromes), and numerous other cellular functions.^4,5^ In essence, iron is an essential nutrient that participates in highly complex biochemical events, vital to human life.

However, the redox activity that makes iron essential for biological functions also imparts significant toxic potential, thus demanding careful regulation of its concentration and distribution to prevent oxidative damage to biological components. Iron deficiency disrupts vital processes such as oxygen transport and DNA synthesis, ultimately resulting in iron deficiency anemia, a condition often marked by persistent fatigue and increased susceptibility to infection.^6,7^ Conversely, excess iron can catalyze the conversion of hydrogen peroxide into highly reactive free radical ions, generating reactive oxygen species (ROS) that cause significant oxidative damage to cellular membranes, proteins, and DNA.^8,9^ Such aberrant oxidative stress is associated with severe pathological conditions, including hemochromatosis,^10^ myocardial hemorrhage,^11^ diabetes,^12^ endocrine toxicity^13^ and heightened susceptibility to neurological disorders such as Parkinson's disease.^14^ The EFSA NDA panel established a safe iron intake level of 40 mg per day for adult.^15^ Taken together, these factors necessitate the development of a highly selective and sensitive Fe^2+^ sensor for precise quantification of labile iron with real-time analytical capability. Despite its biological importance, selective Fe^2+^ detection remains challenging due to its rapid oxidation to Fe^3+^, strong hydration, and interference from competing metal ions.^16^ Therefore, the development of highly selective and sensitive fluorescent probes remains an important research objective.

Numerous established analytical approaches exist for Fe^2+^ detection, including voltammetric and potentiometric methods,^17^ Atomic Absorption Spectroscopy (AAS),^18^ gas chromatography,^19^ High-Performance Liquid Chromatography (HPLC),^20^ electron paramagnetic resonance (EPR)^21^, neutron activation analysis (NAA).^22^ Nevertheless, although these techniques have proven valuable, they exhibit certain limitations, including moderate selectivity and sensitivity, delayed response times, dependence on advanced instrumentation, cost and portability, which may limit their effectiveness for real-time monitoring applications.

Consequently, there is a growing demand for the development of alternative analytical strategies that are selective, cost-effective, sensitive, and accurate.^23^ In response, fluorescent probes have established themselves as precise and high-performance analytical tools which function by translating molecular recognition events into measurable optical signals through variations in fluorescence intensity, emission wavelength thereby offering minimal operational requirements and strong potential for on-site monitoring.

Recently many fluorescent probes have been designed for the detection of ferrous ions. Rattanopas *et al.* designed an indole-based fluorescent sensor for sensing of Fe^2+^ ions. The probe demonstrated measurable fluorescence responses upon binding to Fe^2+^.^24^ Jana *et al.* designed chalcone-based fluorescent sensors for Fe^2+^ detection, exhibiting turn-on fluorescence in aqueous-organic media.^25^ A ratiometric thiophen-imidazoquinazoline fluorescent probe was developed for Fe^2+^ and Fe^3+^ detection, exhibiting dual-emission characteristics.^26^ Enhanced fluorescence in EtOH/H_2_O mixtures was achieved using a spiropyran-based fluorescent probe developed for Fe^2+^ and pH sensing.^27^ Despite these advances, many reported Fe^2+^ fluorescent probes still suffer from certain limitations such as aggregation-caused quenching (ACQ) in aqueous media, small stokes shifts, and limited selectivity in the presence of competing metal ions. Additionally, moderate sensitivity and reduced performance under practical sensing conditions restrict their broader analytical applicability. To address these limitations, we designed and synthesized a pyridine based fluorescent probe with enhanced Fe^2+^ sensing performance, where pyridine units serve as efficient coordination sites for divalent transition metals.^28^ The Lewis-basic pyridine nitrogen enables strong and selective chelation with Fe^2+^, forming stable metal ligand complexes essential for effective sensing.^29^ The incorporation of amide functionalities adjacent to the pyridine core further enhances Fe^2+^ binding through additional nitrogen/oxygen donor sites, thereby improving selectivity and sensitivity over competing metal ions.^30^ To overcome aggregation caused quenching (ACQ), diphenylamine units were incorporated in the sensor molecule. The diphenylamine units adopt a non-planar, propeller-like conformation, which suppresses π–π stacking and restricts intramolecular rotations (RIR) in the aggregated state. This structural arrangement enables the molecule to exhibit aggregation-induced emission (AIE), with a pronounced red shift.^31,32^ Several fluorescent probes have been developed for Fe^2+^ sensing through different quenching pathways, with varying selectivity profiles and detection limits, highlighting the need for further probe development with improved sensing performance (Table S1). To address the common limitations in fluorescent sensors, DPD was rationally designed by incorporating para-amino diphenylamine moieties as strong electron-donating units, forming an effective donor–acceptor–donor (D–A–D) architecture with the electron-deficient pyridine core. The absorption maximum at approximately 330 nm and emission maximum at 412 nm correspond to a stokes shift of approximately 82 nm (Fig. S1). The stokes shift minimizes spectral overlap and self-absorption, which not only improves signal clarity but also contributes to the high sensitivity of the probe in detecting Fe^2+^ ions.^33–35^ Beyond sensitivity and selectivity, recent advances in chemical sensing have emphasized real-time monitoring, quantitative signal interpretation, and reliable performance under practical conditions including ion sensing in biological substrates.^36^ Hence, the practical applicability of DPD was also evaluated using potato samples for real-sample detection of Fe^2+^.

## Experimental section

### Chemicals and reagents

All reagents and chemicals used in this study were obtained from commercial suppliers, including Sigma-Aldrich (USA), Daejung Chemicals & Metals (Korea), Alfa Aesar (UK), and Oakwood Chemicals (USA), and were of analytical grade without further purification. Structural characterization of sensor DPD was performed using ^1^H, ^13^C NMR and DEPT 135 NMR spectroscopy on a Bruker Avance III spectrometer, employing DMSO-d6 as the deuterated solvent. Photoluminescence measurements were carried out using a PerkinElmer LS-45 spectrofluorometer (Hitachi, Japan). Fluorescence emission spectra were recorded with an excitation wavelength of 330 nm, and the maximum emission intensity was observed at 412 nm.

### Synthesis

Pyridine-2,6-dicarbonyl dichloride (DPA chloride) was first prepared by treating pyridine-2,6-dicarboxylic acid (DPA) (3.0 mmol) with SOCl_2_ (2.0 mL) in the presence of catalytic DMF at 80 °C for 12 h. Excess SOCl_2_ was removed under reduced pressure. In a subsequent step *p*-amino diphenylamine (6.0 mmol) and triethylamine (6.0 mmol) in THF were added dropwise to the acid chloride solution and stirred at room temperature for 2 hours. Completion of reaction was observed with the formation of dark green precipitates. After solvent removal, the crude product was filtered using Whatman filter paper and was further purified by column chromatography using petroleum ether/ethyl acetate (3 : 1, v/v). Targeted compound DPD was obtained as yellow crystals in 90% yield (Scheme 1).

**Scheme 1:**
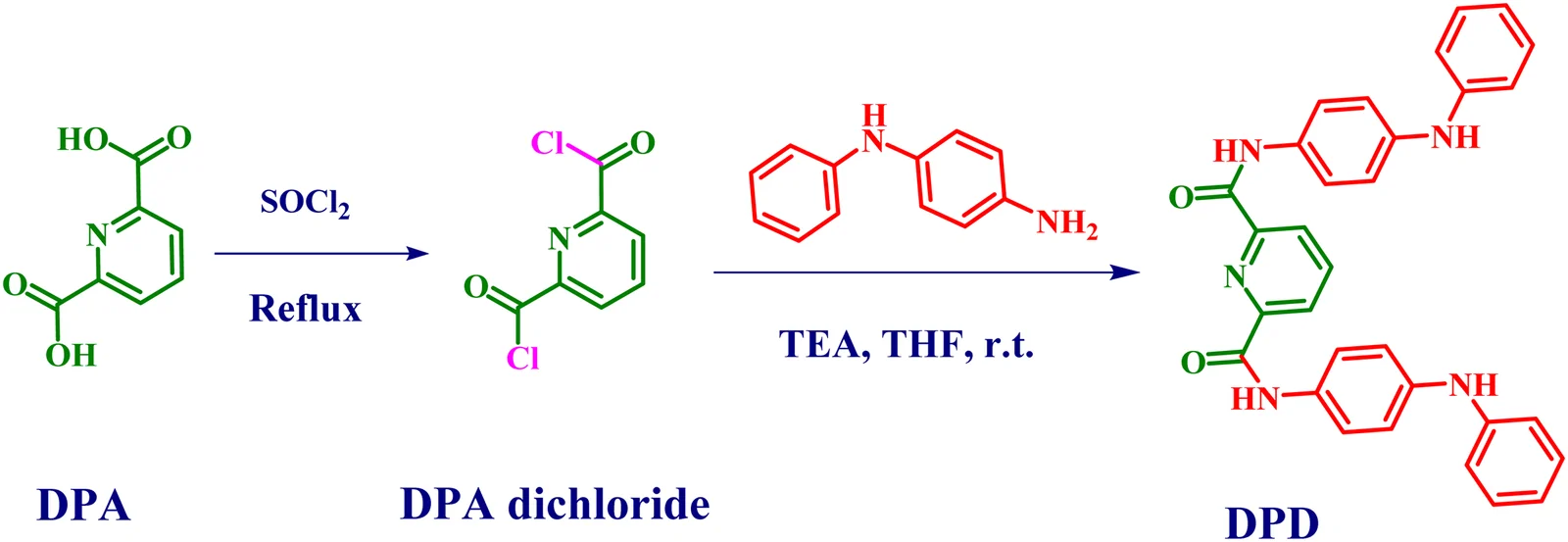
Synthetic route for the preparation of DPD.

Comprehensive NMR characterization (^1^H, ^13^C, and DEPT-135 NMR spectroscopy) was carried out to verify the chemical structure of the synthesized compound DPD. ^1^H NMR shows appearance of a singlet at *δ* 10.92 ppm for –NH protons and absence of NH_2_ signal in 3.0 to 4.0 ppm range confirms the formation of compound (Fig. 1). Furthermore, total number of carbons (31) with carbonyl peak at 161.67 ppm were justified from the ^13^C NMR spectrum (Fig. S2). DEPT-135 analysis was performed to identify quaternary carbons (Fig. S3). Mass spectrometry analysis support the formation of desired structure (Fig. S13). Yield: 90%, ^1^H NMR (400 MHz, DMSO-d_6_) *δ* 10.93 (s, 2H, amide NH), 8.37 (d, *J* = 7.3 Hz, 2H, ArH), 8.27 (t, *J* = 7.4 Hz, 1H, ArH), 7.76 (d, *J* = 8.8 Hz, 4H, ArH), 7.23 (t, *J* = 7.8 Hz, 4H, ArH), 7.16 (d, *J* = 8.8 Hz, 4H, ArH), 7.08 (d, *J* = 7.7 Hz, 4H, ArH), 6.81 (t, *J* = 7.3 Hz, 2H, ArH). ^13^C NMR (100 MHz, DMSO-d_6_) *δ* 161.7 (2 × C), 149.5 (2 × C), 144.1 (2 × C), 140.5 (2 × C), 140.3 (1 × CH), 130.9 (2 × C), 129.7 (4 × CH), 125.4 (2 × CH), 123.2 (4 × CH), 119.9 (2 × CH), 117.6 (4 × CH), 116.7 (4 × CH). DEPT 135 NMR (100 MHz, DMSO-d_6_) *δ* 140.3 (1 × CH), 129.7 (4 × CH), 125.4 (2 × CH), 123.2 (4 × CH), 119.9 (2 × CH), 117.6 (4 × CH), 116.7 (4 × CH). LRMS (ESI): *m*/*z* [M − H]^−^ calculated for C_31_H_24_N_5_O_2_, 498.20; found for C_31_H_24_N_5_O_2_, 498.42.

**Fig. 1:**
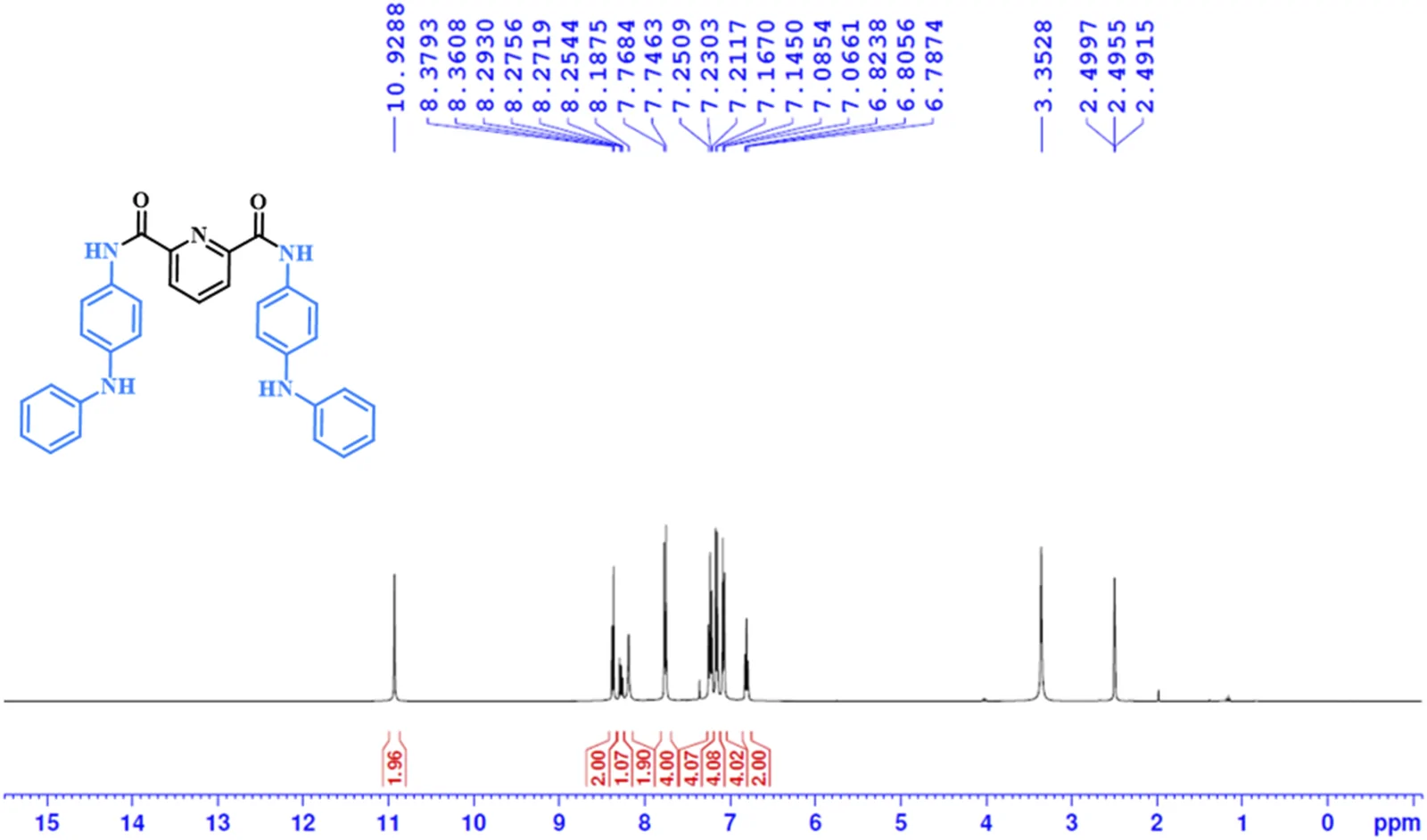
^1^H NMR spectrum of sensor recorded in DMSO-d_6_ at 400 MHz.

### Optical studies

Fluorescence measurements of sensor DPD were carried out in a mixed DMF/H_2_O solvent system (1 : 9, v/v) at an optimized concentration of 30 µM. Emission spectra were recorded upon excitation at 330 nm, and sensor DPD exhibited a maximum fluorescence emission at 412 nm. The fluorescence behavior of DPD was further investigated in DMF by gradually increasing the water content from 0 to 90% (v/v).

To assess the selectivity of sensor DPD, 1 mM stock solutions of various metal salts were prepared, including barium chloride (BaCl_2_), magnesium chloride (MgCl_2_), sodium chloride (NaCl), calcium chloride (CaCl_2_), ferric chloride (FeCl_3_), nickel chloride (NiCl_2_), zinc chloride (ZnCl_2_), tin(ii) chloride (SnCl_2_), ferrous sulfate (FeSO_4_), lithium chloride (LiCl), manganese(ii) chloride (MnCl_2_), chromium(iii) chloride (CrCl_3_), potassium chloride (KCl), copper(i) iodide, copper(ii) chloride (CuCl_2_), mercury(ii) chloride (HgCl_2_). These metal ions were individually spiked into the optimized DPD solution in DMF/H_2_O (1 : 9, v/v), and fluorescence titration experiments were performed with different concentrations of these metal ions. In addition, the fluorescence responses of DPD were examined in the presence of potential interfering species including Cl^−^, SO_4_^2−^, NO_3_^−^, F^−^, CN^−^, OH^−^, and SO_3_^2−^, I^−^ to evaluate possible interference and effects on sensing response.

### Cyclic voltammetry

Cyclic voltammetry (CV) was performed in acetonitrile (ACN) using tetrabutylammonium hexafluorophosphate (TBAPF_6_, 0.1 M) as the supporting electrolyte. A three-electrode cell was used, comprising a glassy carbon disk working electrode, a platinum wire counter electrode, and an Ag/AgNO_3_ (0.01 M) reference electrode. For DPD, the potential was swept from 0 V to +1.3 V, then to −2.3 V, and back to 0 V. The DPD–Fe(ii) complex was scanned from 0 V to +1.3 V, then to −2.4 V, and back to 0 V. All scans were recorded at 100 mV s^−1^. Ferrocene was measured under identical conditions as an internal reference, and its half-wave potential, *E*½ = (*E*_pa_ + *E*_pc_)/2 = 0.100 V, was used to convert all potentials to the ferrocene scale. Oxidation and reduction potentials of DPD and the DPD–Fe complex were determined by cyclic voltammetry. For DPD, the oxidation onset was 0.59 V and the reduction onset was −2.25 V, whereas the complex showed an oxidation onset at 0.31 V and no reduction peak. Scan-rate dependence was evaluated over 10–200 mV s^−1^, and linear regression of the anodic peak current against the square root of scan rate gave *R*^2^ = 0.963, consistent with diffusion-controlled behaviour.

### DFT methodology

Density Functional Theory (DFT) was employed to obtain computational insights into the sensing behavior of the DPD sensor using the Gaussian 09 software package.^37^ All quantum chemical calculations associated with the selected DPD fluorophore were performed in Gaussian 09, while molecular architecture and electronic outputs were visualized through Gauss view 5. The ground-state molecular geometry of the DPD sensor was fully optimized at the B3LYP (Becke's three-parameter hybrid exchange functional) level in conjunction with the 6-311G(d,p) basis set.^38^ The computational calculations were performed using the singlet spin state, the LANL2DZ basis set for Fe, and a gas-phase model without solvent effects. Frequency calculations were performed for the optimized structure, and the calculated vibrational frequencies and corresponding infrared intensities are provided in Table S2 of the SI. The optimized geometry of the DPD sensor was subsequently employed for all electronic structure and interaction energy calculations (Fig. S4). Subsequently, the binding interaction energy between DPD and Fe^2+^ was evaluated using the ωB97XD functional, owing to its superior capability in describing long-range electron correlation and noncovalent interactions primarily arising from London dispersion forces.^39^ The interaction energy was evaluated with consideration of the basis set superposition error (BSSE) correction. The calculated energy of −1743.516356744 kcal mol^−1^ was obtained for the specified computational model.

To elucidate the nature of weak intermolecular forces governing complex formation, Multiwfn 3.8 software was employed for non-covalent interaction (NCI) analysis together with QTAIM (Quantum Theory of Atoms in Molecules) topological investigations.^40^ The resulting reduced density gradient isosurfaces and bond critical point analyses were further rendered and interpreted using VMD software for enhanced structural visualization.^41^ Furthermore, comprehensive orbital-level analyses, including Natural Bond Orbital (NBO), Frontier Molecular Orbital (FMO), and Density of States (DOS) studies, were carried out for the electronic characteristics of the sensing assembly. Through NBO calculations, both stabilization energies and the magnitude of charge delocalization between interacting fragments were quantified.^42^ Such intramolecular and intermolecular charge redistribution substantially modulates the intrinsic electronic profile of DPD, leading to pronounced variations in conductivity, resistance, and band-gap energy upon interaction with Fe^2+^.

## Result and discussion

### Chemistry of DPD

The sensor was rationally designed based on a donor–acceptor–donor (D–A–D) molecular scaffold, in which the pyridine-2,6-dicarboxamide unit functions as a central electron-deficient acceptor, while the terminal *p*-amino diphenylamine groups act as strong electron donors (Fig. S5). The synthesis was accomplished through activation of pyridine-2,6-dicarboxylic acid using thionyl chloride as a chlorination agent followed by amide coupling with *para*-substituted diphenylamine affording a symmetric *N*^2^,*N*^6^-bis(4-(phenylamino)phenyl)pyridine-2,6-dicarboxamide framework. The rigid pyridine core provides favorable spatial arrangement and introduces a nitrogen donor site, whereas the two amide linkages supply carbonyl oxygen atoms capable of metal coordination while simultaneously facilitating electronic communication between donor and acceptor segments. This structural arrangement establishes an extended π-conjugated pathway that supports intramolecular charge transfer (ICT) from the peripheral donor units toward the central acceptor upon photoexcitation.

Upon excitation, electron density is transferred from the diphenylamine-based donor moieties to the pyridine-dicarboxamide acceptor core, generating a stabilized ICT excited state. This donor-to-acceptor charge migration is responsible for the broad emission band and the stokes shift of 82 nm which effectively minimizes self-absorption and enhances detection sensitivity. The D–A–D configuration amplifies environmental responsiveness, making the sensor highly sensitive to changes in polarity, aggregation state, and metal coordination. The twisted, non-planar geometry of the phenylamine donors effectively prevents strong π–π stacking interactions, favoring the formation of emissive aggregates rather than aggregation-caused quenching (ACQ).

For metal ion sensing studies conducted at 90% water content, the sensor exhibits selective fluorescence quenching in the presence of Fe^2+^ ions. This response arises from interaction of Fe^2+^ with the pyridine nitrogen atom and the amide carbonyl oxygen atoms. Metal binding alters the electronic distribution of the molecule and suppresses the donor-to-acceptor charge transfer, leading to efficient fluorescence quenching. Actually, Fe^2+^ interaction with sensor DPD can promote excited-state electron transfer (energy transfer processes) due to the accessible d-orbitals along with paramagnetic nature of Fe^2+^. This electron transfer facilitates non-radiative excited-state relaxation, further contributing to quenching of fluorescence emission. The pronounced selectivity toward Fe^2+^ can be attributed to its suitable ionic radius, preferred coordination geometry, and strong affinity for mixed nitrogen – oxygen donor ligands. Collectively, the deliberate integration of a charge-transfer active conjugated framework and aromatic moieties promoting aggregation accounts for the observed aggregation-induced emission. Sensor DPD with a well-defined available metal-binding sites toward Fe^2+^ offers selective fluorescence quenching in aqueous environments.

### Fluorescence intensity optimization

To investigate the concentration dependent photophysical response of the DPD sensor, its absorption and fluorescence responses were examined over a concentration range of 10 to 30 µM. The optical performance of fluorescent sensors is strongly influenced by the inner filter effect (IFE), which is directly related to fluorophore concentration therefore, selecting an appropriate concentration is essential to obtain optimal emission intensity and reliable analytical performance. At elevated concentrations, DPD molecules tend to approach each other closely, leading to π–π stacking interactions. Under these conditions, excitation light is not uniformly absorbed by all fluorophore molecules, and a portion of the emitted photons is reabsorbed by neighboring molecules, resulting in emission quenching due to IFE.

As the concentration of DPD increased from 10 to 30 µM, both absorbance and fluorescence intensity showed gradual enhancement, indicating efficient excitation and emission processes within this concentration window. However, further increase in concentration led to a decline in emission intensity, which can be attributed to the dominance of inner filter effects at higher fluorophore loadings. The DPD sensor exhibited two absorption bands, one of high energy with a maximum absorption at *λ*_abs_ = 285 nm while lower energy band with absorption maximum *λ*_abs_ = 330 nm in THF (Fig. S6a). Meanwhile fluorescence studies also revealed two emission bands first emission band with a maximum emission at *λ*_em_ = 412 nm while the highest fluorescence intensity of second band was observed at a concentration of 30 µM with an emission maximum at *λ*_em_ = 530 nm in DMF (Fig. S6b). Therefore, 30 µM was selected as the optimum concentration for further fluorescence sensing studies, as it provides strong emission intensity without noticeable spectral distortion. In addition, the comparison of absorption and emission spectra showed a stokes shift of 82 nm (330 nm excitation, 412 nm emission), providing spectral separation that may help minimize reabsorption and improve fluorescence signal clarity, ensuring reliable fluorescence measurements.

### Aggregation studies

The aggregation-induced photophysical behavior of the sensor was explored using UV-visible absorption spectroscopy in THF/H_2_O mixtures and fluorescence spectroscopy in DMF/H_2_O systems. In donor–acceptor–donor (D–A–D) chromophores, UV-vis spectra often shows two distinct absorption bands, with the high-energy band arising from localized π → π* transitions and a lower-energy band due to intramolecular charge transfer (ICT).^43,44^ In line with previous reports on D–A–D systems, compound DPD displays two well-defined absorption bands. The high-energy UV band (285 nm) and lower-energy band (334 nm) (Fig. 2a). The former band (285 nm) shows minimal variation with solvent composition, while the lower-energy band (330 nm) exhibits pronounced broadening and red shift, with increasing water content. These spectral changes are consistent with solvent-dependent photophysical behavior and aggregation, with possible contributions from intramolecular charge transfer (ICT), as reported for related systems. In the lower-energy absorption band, the *λ*_max_ gradually shifts from 334 nm to 339 nm as the water fraction increases from 0% to 70%, followed by a more pronounced shift to 375 nm at 80–90% water fraction. The significant change at higher water content is likely due to enhanced intramolecular charge-transfer (ICT). ICT can occur in conjugated donor–acceptor systems, in which photoexcitation may induce a redistribution of electron density from the donor to the acceptor moiety. In polar, high-water-content environments, the excited state may be further stabilized by the surrounding medium, while reduced solubility can promote molecular aggregation. Depending on the intermolecular arrangement, such aggregation may contribute to changes in the electronic absorption characteristics. In particular, J-type aggregation can produce red-shifted absorption features, while enhanced ICT character may also contribute to bathochromic shifts in absorption or emission spectra.^45,46^

**Fig. 2:**
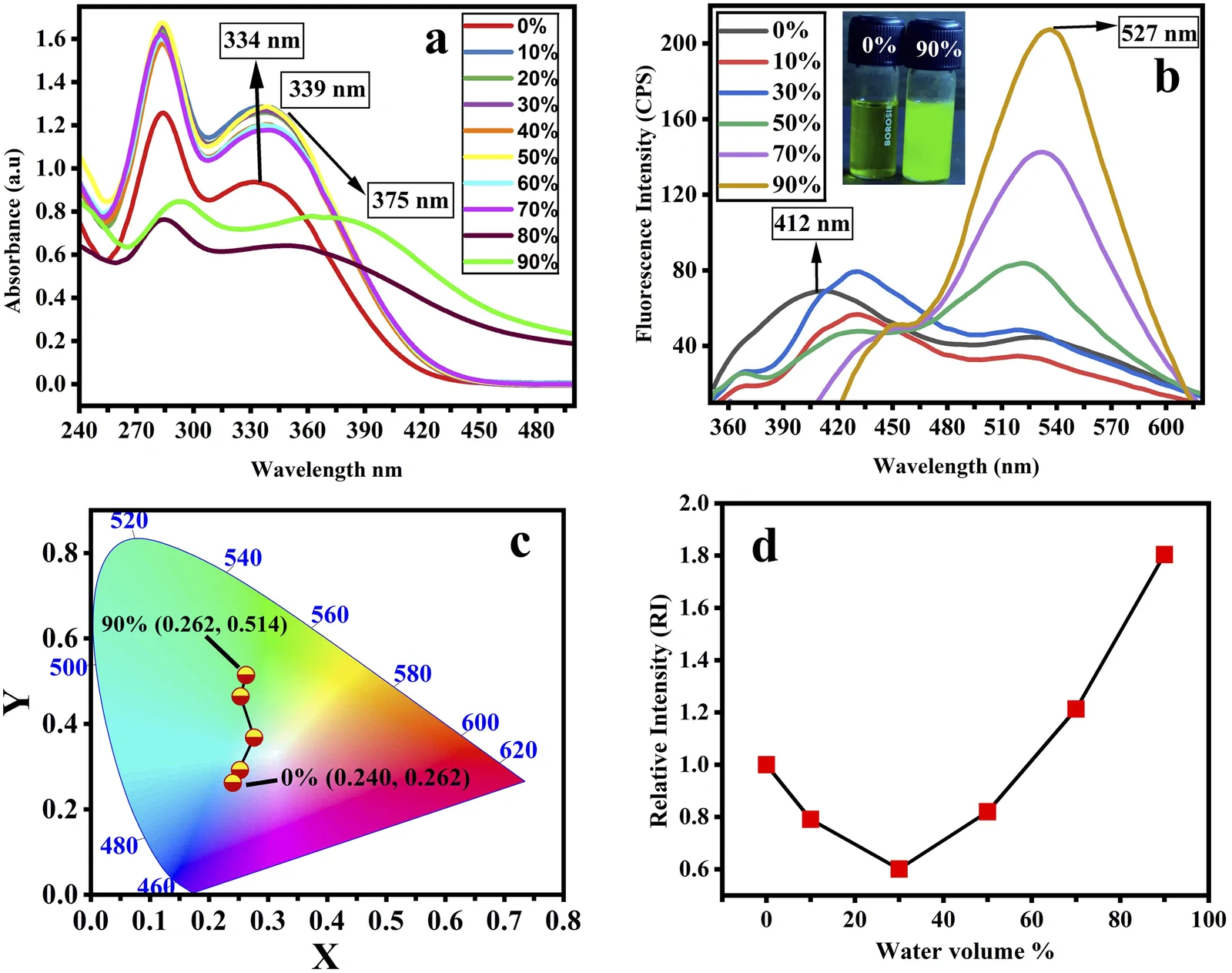
UV-visible absorption spectra of the sensor recorded at different water fractions (0–90%, v/v) (a). Fluorescence emission spectra of the sensor at varying water fractions (0–90%, v/v) (b). CIE 1931 chromaticity diagram illustrating the change in emission color with increasing water fraction (c). Plot of relative fluorescence intensity as a function of water fraction (d).

The fluorescence behavior of sensor was examined in mixed solvents of DMF/water (Fig. 2b). In pure DMF (0% water), the emission spectrum is dominated by a band at *λ*_1_ = 412 nm, assigned to the locally excited (LE) monomeric state, which arises from radiative decay of the lowest singlet excited state (S_1_ → S_0_)^47^ and originates primarily from a π–π* transition of the monomeric species. In 90% water fraction, a weak but distinct long-wavelength emission band at *λ*_2_ = 527 nm is observed, consistent with an partially intramolecular charge-transfer (ICT) type excited state, arising from a relaxed excited state (Fig. 2).^47^ As the water fraction is increased, the LE emission gradually diminishes, while the long wavelength band significantly enhanced intensity. This behavior reflects water-induced aggregation, which restricts intramolecular rotations and vibrations, reduces non-radiative decay, and channels the excited-state population into the aggregated emissive state. The increase in fluorescence intensity at 90% water fraction demonstrates aggregation-induced emission (AIE) behavior with an emission maximum of 527 nm, while the nearly complete quenching of the monomeric band indicates that most molecules now participate in the formation of aggregates. In addition, a CIE chromaticity diagram was constructed to visualize the solvent-dependent changes in the emission color of the DPD sensor (Fig. 2).

The emission coordinates gradually move from the blue region toward the green region, indicating a bathochromic shift in the fluorescence emission. The formation of aggregates upon the addition of a poor solvent (water) to the DPD solution in DMF was investigated by dynamic light scattering (DLS) measurements. DLS analysis was employed to monitor changes in the hydrodynamic size of DPD under different solvent compositions. In pure DMF, the DPD molecules exhibited an average particle size of 328.7 nm, suggesting the presence of associated species under these conditions. Upon increasing the water fraction to 90%, the average hydrodynamic particle size increased markedly to 1064 nm, indicating enhanced aggregation and particle growth in the water-rich medium (Fig. S7). Based on these observations, a DMF/water mixture with a volume ratio of 1 : 9 (v/v) was selected as the optimized medium for subsequent fluorescence sensing studies.

### Sensing studies

#### Sensing of Fe^2+^ ions

The fluorescence sensing behavior of sensor DPD toward ferrous ions (Fe^2+^) was systematically investigated in a mixed solvent system of DMF : H_2_O (1 : 90, v/v). To evaluate selectivity, solutions of various competing metal ions were prepared in the range of 0–1000 µM and individually spiked into a fixed concentration of DPD (30 µM). The examined metal ions include, barium(ii) (Ba^2+^), magnesium(ii) (Mg^2+^), sodium(i) (Na^+^), calcium(ii) (Ca^2+^), fluoride (F^−^), iron(iii) (Fe^3+^), nickel(ii) (Ni^2+^), zinc(ii) (Zn^2+^), tin(ii) (Sn^2+^), iron(ii) (Fe^2+^), lithium(i) (Li^+^), manganese(ii) (Mn^2+^), chromium(iii) (Cr^3+^), copper(ii) (Cu^2+^), copper(i) (Cu^+^), and mercury(ii) (Hg^2+^), to evaluate its selectivity toward Fe^2+^.

Notably, fluorescent sensor DPD exhibited negligible emission changes in the presence of most competing ions, confirming its strong tolerance against common mono-, di-, and trivalent metal ions. In contrast, Fe^2+^ induced a pronounced decrease in fluorescence intensity (Fig. S8), demonstrating a highly selective “turn-off” response. This selective quenching behavior supports the preferential interaction of Fe^2+^ with the emissive state of DPD, likely due to stronger coordination affinity and excited-state deactivation pathways promoted by Fe^2+^.

To quantify the response, fluorescence titration experiments were carried out by gradually increasing the concentration of Fe^2+^ (0–100 µM) in the DPD solution. DPD displayed an intense emission band, which decreased progressively upon Fe^2+^ addition, confirming concentration dependent quenching (Fig. 3a). The strongest fluorescence quenching was achieved at the maximum Fe^2+^ concentration examined, demonstrating highly effective signal attenuation even at trace concentrations. The quenching efficiency and binding strength were evaluated using the Stern–Volmer relationship:*I*_0_/*I* = 1 + *K*_SV_[Q]where *I*_0_ is the fluorescence intensity of free DPD, *I* is the intensity after Fe^2+^ addition, *K*_SV_ is the Stern–Volmer constant, and [Q] represents the concentration of Fe^2+^. A Stern–Volmer plot of *I*_0_/*I versus* Fe^2+^ concentration showed a clear response trend, indicating strong interaction between DPD and Fe^2+^. A linear Stern–Volmer relationship was obtained over the investigated concentration window, indicating a consistent quenching mechanism without any noticeable deviation from linearity (Fig. S9). The limit of detection (LOD) for Fe^2+^ was calculated using the standard expression:LOD = 3*σ*/*S*

**Fig. 3:**
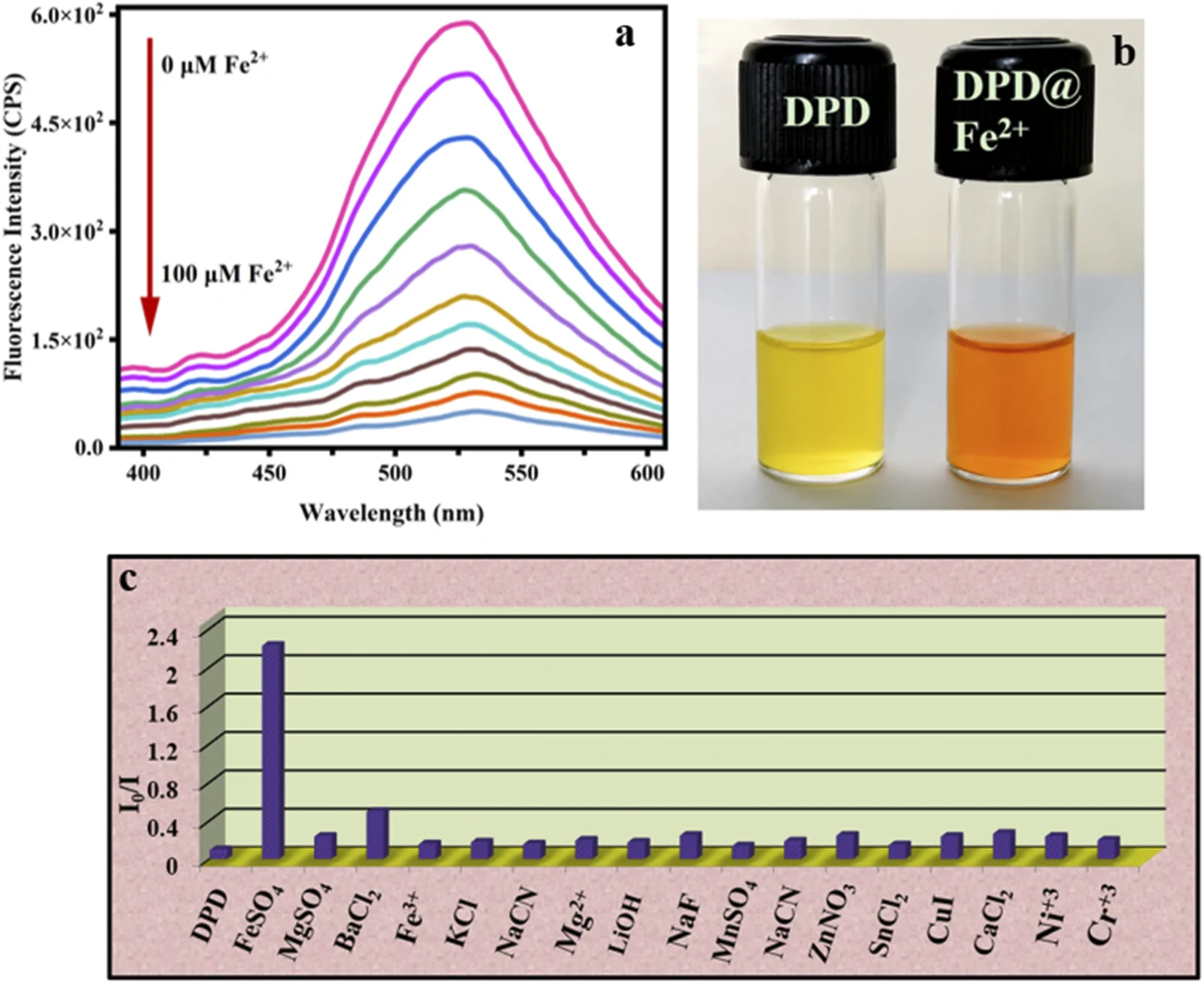
Fluorescence quenching of DPD with increasing Fe^2+^ concentration (0–100 µM) (a). Colorimetric response of DPD before and after addition of Fe^2+^ under daylight (b). Comparative 2D Stern–Volmer (SV) plot illustrating the selectivity of DPD toward Fe^2+^ over various analytes (1.0 mM concentrations) (c).

Taking *σ* = 0.0926 (standard deviation/standard error of intercept shown on the graph), LOD = 4.10 µM where *σ* represents the standard deviation of blank measurements and *S* is the slope of the calibration plot. The obtained LOD demonstrates that DPD can detect Fe^2+^ at micromolar levels, highlighting its suitability for trace analysis. The fluorescence quenching efficiency was calculated using the equation:Quenching efficiency (%) = [(*F*_0_ − *F*)/*F*_0_] × 100where *F*_0_ is the fluorescence intensity of the free sensor molecules and *F* is the fluorescence intensity after addition of Fe^2+^. The fluorescence intensity of the free probe decreased from 588.74 to 110.50 upon addition of Fe^2+^, corresponding to a fluorescence quenching efficiency of approximately 81.2% (Fig. S10).

To further support interaction between DPD and Fe^2+^, dynamic light scattering (DLS) analysis was performed to monitor changes in particle size distribution before and after Fe^2+^ addition. The aggregated DPD system exhibited an average hydrodynamic diameter of 1064 nm (Fig. S7b), which increased markedly to 1345 nm upon Fe^2+^ incorporation (Fig. S11). The observed increase in hydrodynamic size suggests enhanced aggregation and possible interactions between DPD and Fe^2+^, potentially resulting in larger associated assemblies. Such aggregation is consistent with the observed fluorescence quenching, as the generation of larger supramolecular clusters can promote non-radiative relaxation pathways and consequently suppress emission intensity. Overall, the combined fluorescence selectivity profile, titration-dependent quenching, Stern–Volmer analysis, and supportive DLS evidence confirm that DPD functions as a selective fluorescent chemosensor for Fe^2+^ ions, operating through a robust turn-off sensing mechanism in aqueous-rich media.

### Sensing mechanism

In free state, photoexcitation of DPD leads to the formation of an intramolecular charge transfer (ICT) excited state. Now electrons in the highest occupied molecular orbital (HOMO), largely localized on the phenylamino donor groups, are promoted to the lowest unoccupied molecular orbital (LUMO). Because these donor and acceptor subunits are rigidly conjugated through planar amide linkages, the excited electron and the corresponding hole remain spatially delocalized across the π-system, resulting in an emissive singlet excited state with a well-defined energy gap. This results in strong fluorescence emission at a characteristic wavelength that reflects the HOMO–LUMO gap of the free ligand.

Upon addition of Fe^2+^, selective coordination occurs *via* the pyridine nitrogen atom and the two amide carbonyl oxygens, creating a pre-organized N, O chelation pocket. This coordination engages the lone pairs that previously contributed to the π-system, altering the local electronic environment of the fluorophore without strongly perturbing the overall π-conjugation. The frontier orbital energy gap of the conjugated system remains largely unchanged; the emission maximum remains constant upon increasing concentration of Fe^2+^.

The profound reduction in emission intensity, however, reflects the activation of a new non-radiative deactivation pathway upon excitation of the probe bound Fe^2+^ interaction. In this complex, transition metal center of the Fe^2+^ ion with accessible empty d-orbitals functions as an effective electron acceptor in a photoinduced electron transfer (PET) process. Upon photon absorption, an electron is promoted from the ground state HOMO to the excited state LUMO of the fluorescent sensor DPD. Once in the excited state, instead of relaxed radiatively back to the ground state, the electron can now transfer from the fluorophore's excited orbital into vacant Fe^2+^ associated orbitals (*e.g.*, Fe^2+^ 3d orbitals) through a thermodynamically favorable PET process (Fig. 4). This excited-state electron transfer competes effectively with radiative decay, resulting in efficient quenching of fluorescence intensity.

**Fig. 4:**
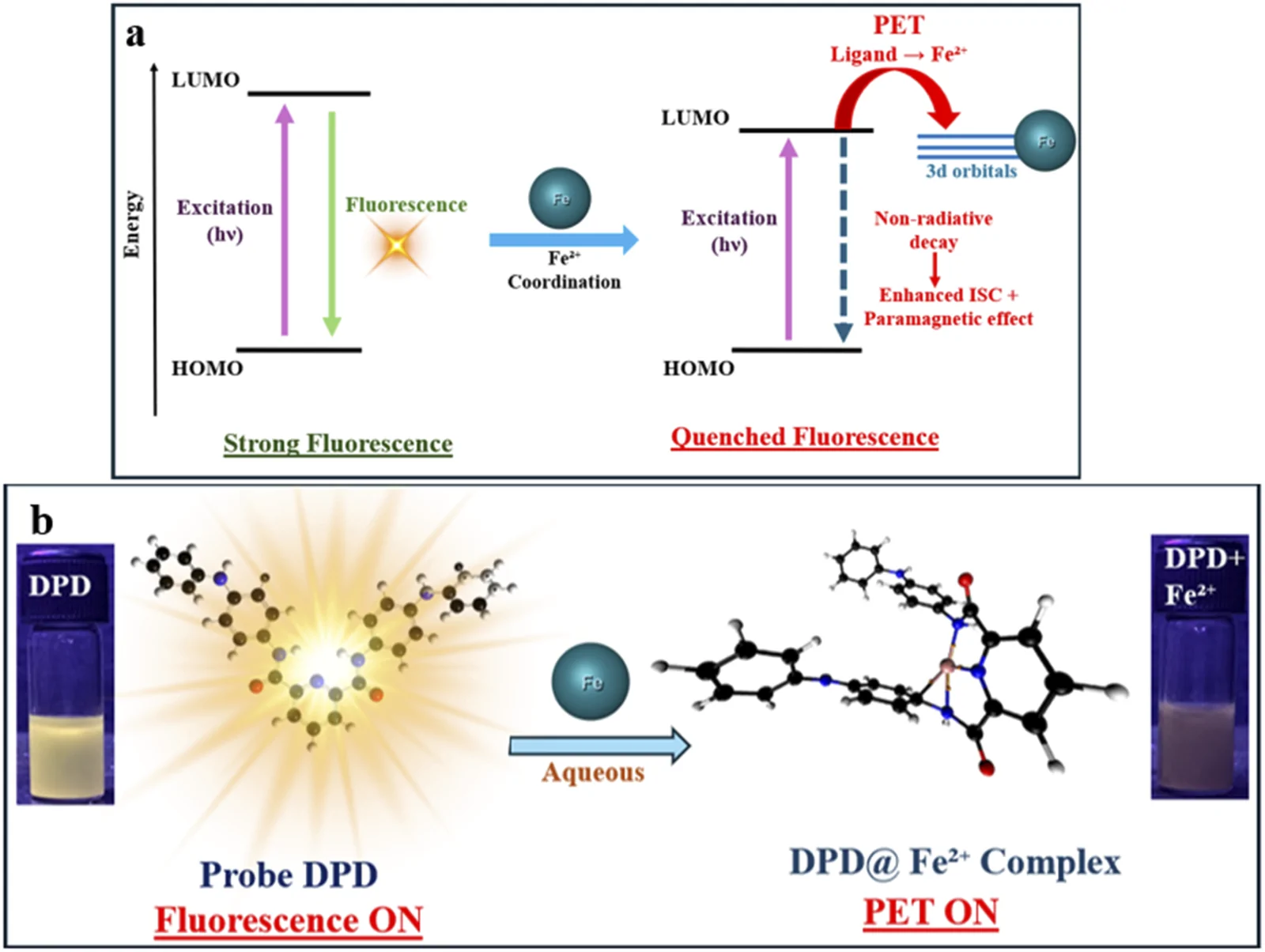
Proposed fluorescence sensing mechanism of DPD toward Fe^2+^. (a) HOMO–LUMO energy level diagram illustrating the proposed fluorescence quenching mechanism. (b) Schematic illustration of the fluorescence ON–OFF sensing process upon Fe^2+^ coordination.

In addition to PET, the paramagnetic nature of high-spin Fe^2+^ (with unpaired electrons) further enhances non-radiative decay by increasing spin–orbit coupling. This facilitates intersystem crossing from the excited singlet state to non-emissive triplet state manifolds, accelerating non-radiative relaxation and amplifying quenching efficiency (Fig. 4). The coexistence of PET and paramagnetic relaxation explains both the intensity loss and the high sensitivity of sensor DPD toward Fe^2+^. To further investigate the interaction between DPD and Fe^2+^, ^1^H NMR spectra were recorded before and after the addition of Fe^2+^. The amide NH resonance remained unchanged, and the aromatic proton signals in the 8.3–6.8 ppm region exhibited only minor chemical shift variations of approximately 0.03 ppm, accompanied by broadening and overlap of some resonances. The negligible chemical shift variations and unchanged NH signal indicate that the molecular structure of DPD remains essentially intact, without significant coordination with Fe^2+^. The observed peak broadening may be attributed to the paramagnetic nature of Fe^2+^. Collectively, these observations suggest that the interaction between DPD and Fe^2+^ may involve weak noncovalent interactions rather than direct coordination, with the overall structure of the DPD molecule remaining essentially unchanged (Fig. 5).

**Fig. 5:**
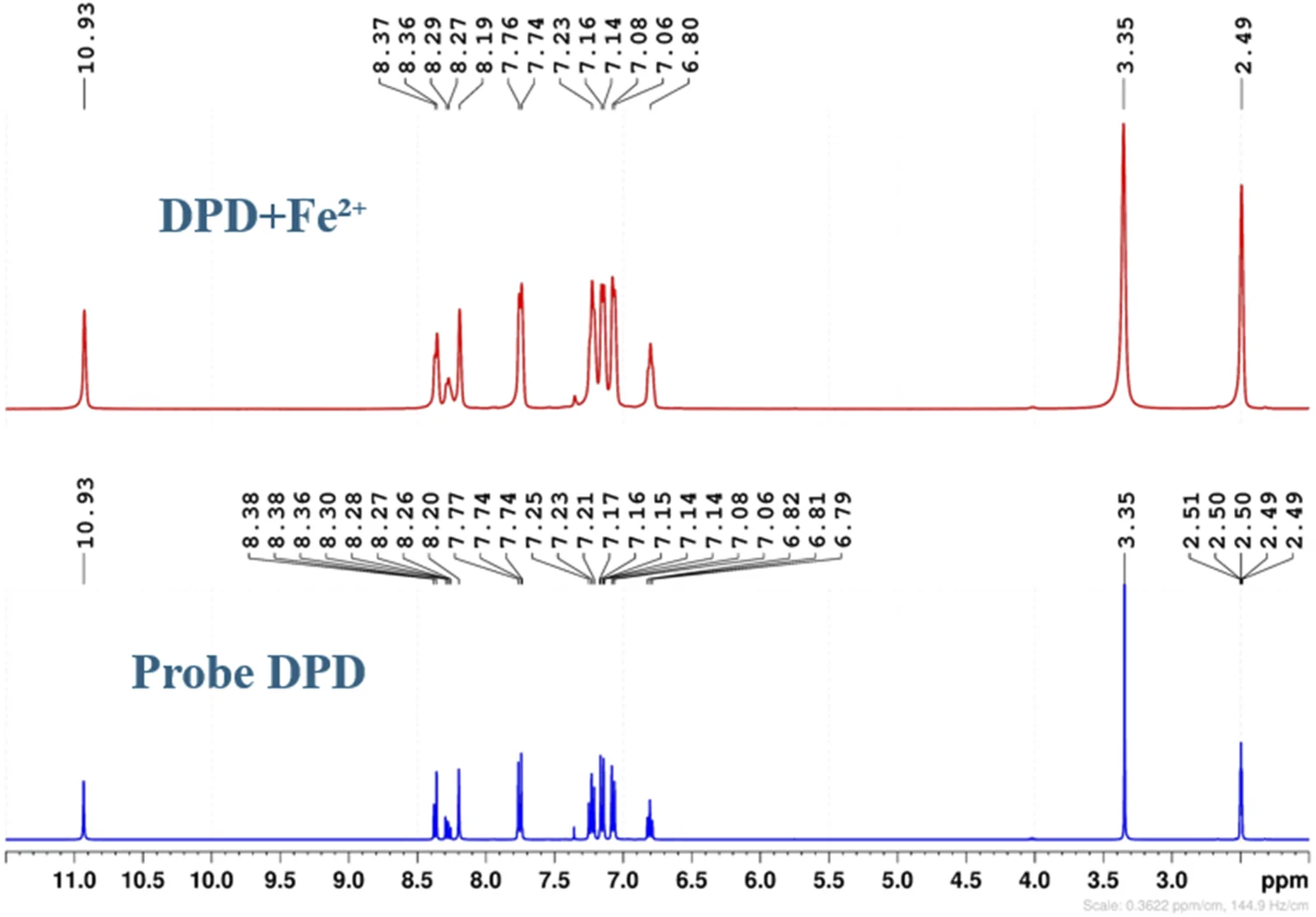
Stacked ^1^H NMR spectra of free DPD sensor and complex (Fe@DPD), showing chemical shift changes upon addition of Fe^2+^ into DPD solution in DMSO-d_6_.

FTIR titration analysis was performed alongside NMR titration experiment to study the functional groups in fluorescent sensor DPD and to confirm the interaction with Fe^2+^ ions. The FTIR spectrum of free DPD showed distinctive absorption bands at 3365.98 cm^−1^ and 3273.85 cm^−1^ for N–H stretching vibrations of the amide and secondary amine functional groups, respectively. Band at 3046.63 cm^−1^ is attributed to aromatic sp^2^ C–H stretching. The strong absorption bands at 1676.85 cm^−1^ and 1652.28 cm^−1^ are assigned to amide carbonyl (C=O) stretching vibrations, while the bands at 1586.76 and 1519.19 cm^−1^ correspond to aromatic C=C/C=N stretching and N–H bending, respectively. The band at 1318.55 cm^−1^ was attributed to C–N stretching. Upon interaction with Fe^2+^, the carbonyl stretching band shifted from 1676.85 to 1648.18 cm^−1^, accompanied by slight shifts of the N–H bending and C–N stretching bands to 1517.15 and 1316.50 cm^−1^, respectively (Fig. S12). These spectral changes suggest the coordination of the amide carbonyl oxygen and nitrogen donor sites with Fe^2+^, confirming the formation of the (DPD@Fe^2+^) complex.

LC-MS analysis was also performed to investigate the interaction between DPD and Fe^2+^. In negative electrospray ionization mode, free fluorescent sensor DPD showed a peak at *m*/*z* 498.42, assigned to [DPD–H]^−^, which agrees with its molecular mass of 499.20 g mol^−1^ (Fig. S13). Upon addition of Fe^2+^, additional peaks were observed a new peak appeared at *m*/*z* 596.33, tentatively assigned to the [DPD + HSO_4_]^−^ adduct, while the peak at *m*/*z* 650.25 is assigned to [Fe(DPD–2H)(HSO_4_)]^−^. The appearance of this new peak provide complementary evidence for the interaction between DPD and Fe^2+^ and formation of the DPD@Fe^2+^ complex during LC-MS titration experiment (Fig. S14).

### Interference analysis

To evaluate the selectivity of sensor DPD toward Fe^2+^, its fluorescence response was systematically examined in the presence of a series of potentially competing species, including common inorganic anions (OH^−^, CN^−^, I^−^, F^−^, SO_4_^2−^, NO_3_^−^) under identical experimental conditions (Fig. S15). The sensor exhibited negligible variation in fluorescence intensity upon addition of these interfering species, with emission profiles largely overlapping that of the free sensor. In sharp contrast, the introduction of Fe^2+^ (as FeSO_4_) resulted in a pronounced quenching of fluorescence intensity, clearly distinguishing it from all other tested analytes.

The practical stability of the DPD sensor was evaluated by studying the effects of temperature, UV irradiation, and response time. The fluorescence intensity of the DPD@Fe^2+^ system remained relatively stable over the temperature range of 10–70 °C, indicating limited temperature dependence (Fig. S16). Photostability studies under continuous UV irradiation showed only a slight change in fluorescence intensity with time, confirming minimal photobleaching (Fig. S17). Furthermore, the fluorescence quenching occurred rapidly after the addition of Fe^2+^ and remained stable, demonstrating a fast and consistent response (Fig. S18).

### Cyclic voltammetry

Cyclic voltammetry (CV) was employed to investigate the redox behavior and estimate the frontier molecular orbital energies of DPD in acetonitrile (ACN). Ferrocene/ferrocenium (Fc/Fc^+^) was used as an internal electrochemical reference, and its half-wave potential was taken as +0.100 V under experimental condition (Fig. S19a). The half-wave potential is determined from the anodic and cathodic peak potentials using following equation:*E*_1/2_ = (*E*_pa_ + *E*_pc_)/2 = 0.100 V

Ferrocene is widely used as an internal reference in nonaqueous electrochemistry because it helps account for variations in reference-electrode potentials.^48^

### Electrochemical studies of DPD

The CV profile of free DPD exhibited an anodic oxidation peak at approximately +0.59 V and a cathodic reduction peak at approximately −2.25 V. The corresponding onset potentials were estimated as +0.45 V for oxidation and −2.05 V for reduction (Fig. 6a). These onset potentials were determined graphically using the tangent method. This approach is commonly used to estimate oxidation and reduction onsets for calculating electrochemical frontier orbital energies. The onset potential, rather than the peak potential, were used in the calculations.^49^ Using these onset potentials and the Fc/Fc^+^ reference potential of +0.100 V, the electrochemical HOMO and LUMO energies of DPD were estimated to be −5.15 and −2.65 eV, respectively, yielding an electrochemical energy gap of 2.50 eV. These values provide an estimate of the redox-derived frontier orbital energy levels of DPD. Cyclic voltammetry measurements were performed at scan rates ranging from 10 to 100 mV s^−1^ to investigate the redox behavior and kinetics of DPD (Fig. S19b). The anodic and cathodic peak currents increased with increasing scan rate. The linear relationship between the anodic peak current and the square root of the scan rate (*R*^2^ = 0.9631) was stronger than that with the scan rate itself (*R*^2^ = 0.9215), suggesting a predominant diffusion-controlled contribution to the electrochemical process (Fig. S20).

**Fig. 6:**
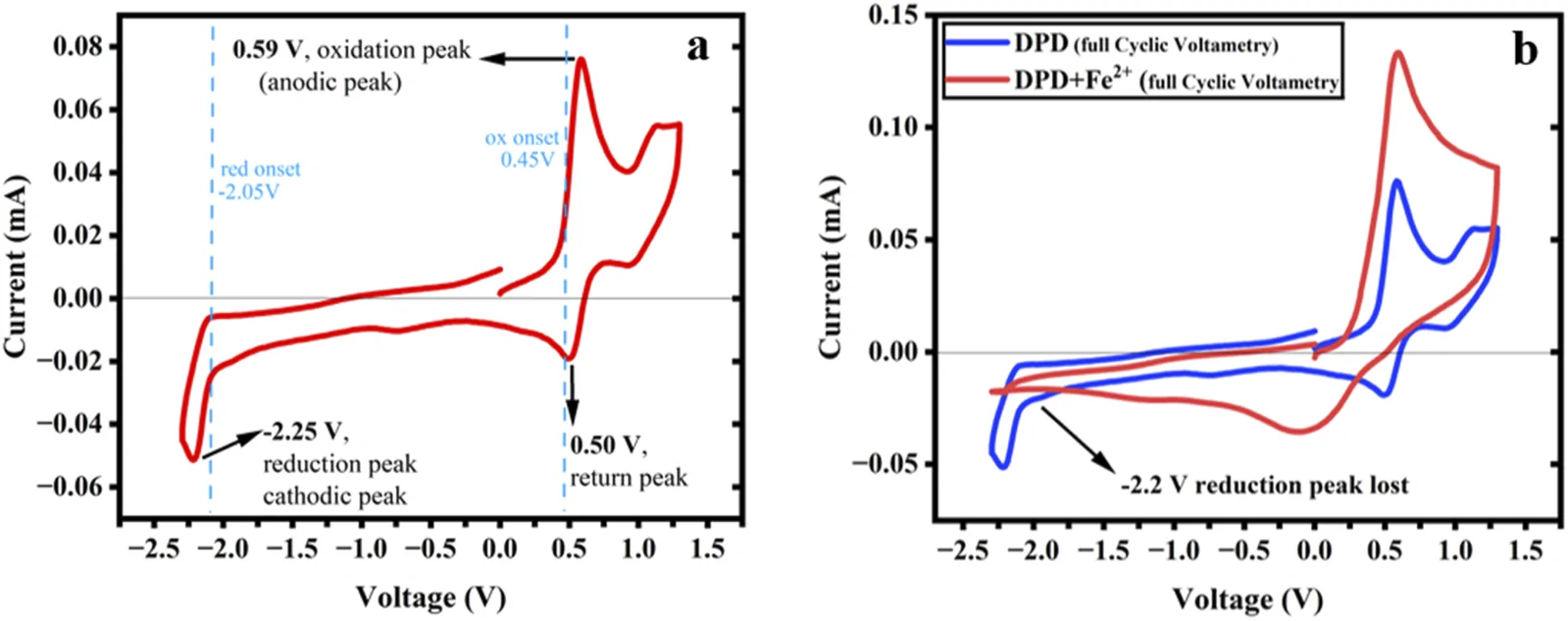
(a) CV profile of free DPD with peak and onset potentials. (b) Comparison of DPD and DPD in the presence of Fe^2+^, showing the disappearance of the reduction peak.

### DPD–Fe^2+^ electrochemical comparison

The oxidation peak observed at approximately +0.59 V is tentatively attributed to the diphenylamine moieties, in which the nitrogen lone pairs are conjugated with the adjacent aromatic rings, facilitating electron donation during oxidation. The reduction peak at approximately −2.25 V may be associated with the pyridine-containing core, which can participate in electron uptake. Upon the addition of Fe^2+^, the reduction peak disappeared, suggesting that interaction between Fe^2+^ and the pyridine nitrogen alters the electronic environment of the reduction site, making it no longer electrochemically accessible under the experimental conditions. This change may arise from the involvement of the pyridine nitrogen lone pair in metal-ion interaction, which modifies the electron-accepting characteristics of the pyridine ring. The CV profiles of free DPD and DPD in the presence of Fe^2+^ were compared to investigate changes in the probe's redox behavior upon interaction with the metal ion (Fig. 6b).

### Computational studies

#### FMO and DOS

To investigate the sensing behavior and electronic response of the sensor, frontier molecular orbital (FMO) analysis was performed before and after interaction with Fe^2+^. The HOMO–LUMO energy gap serves as a key indicator of electronic properties; a larger gap corresponds to higher stability and lower conductivity, whereas a reduced gap reflects enhanced charge transfer, increased chemical reactivity, and improved sensitivity. For the isolated DPD sensor, the HOMO and LUMO energies were calculated as −5.26 eV and −2.03 eV, respectively, with an energy gap of 3.23 eV, indicating moderate stability and limited electronic mobility. Upon interaction (DPD@Fe), a significant shift in energy levels was observed, with HOMO and LUMO values of −10.07 and −9.23 eV, respectively, resulting in a markedly reduced energy gap of 0.84 eV (Fig. S21). This decrease in the band gap indicates enhanced electronic conductivity and strong interaction between DPD and Fe^2+^, attributed to effective orbital overlap and charge transfer between the sensor and the metal center. The HOMO is mainly localized over the π-framework of DPD, while the LUMO shows significant contribution from the metal center, confirming a donor–acceptor interaction and facilitating electron delocalization across the system. Such narrowing of the energy gap is characteristic of highly sensitive sensing systems.

Further insight was obtained through density of states (DOS) analysis (Fig. 7). The pristine state exhibits well-separated occupied and unoccupied states (Fig. 7a), whereas the DPD@Fe complex shows broadening of energy levels and the emergence of new states near the Fermi level, indicating strong orbital hybridization (Fig. 7b). The shift of electronic states toward the Fermi level further supports enhanced conductivity and faster electron transfer upon complex formation.

**Fig. 7:**
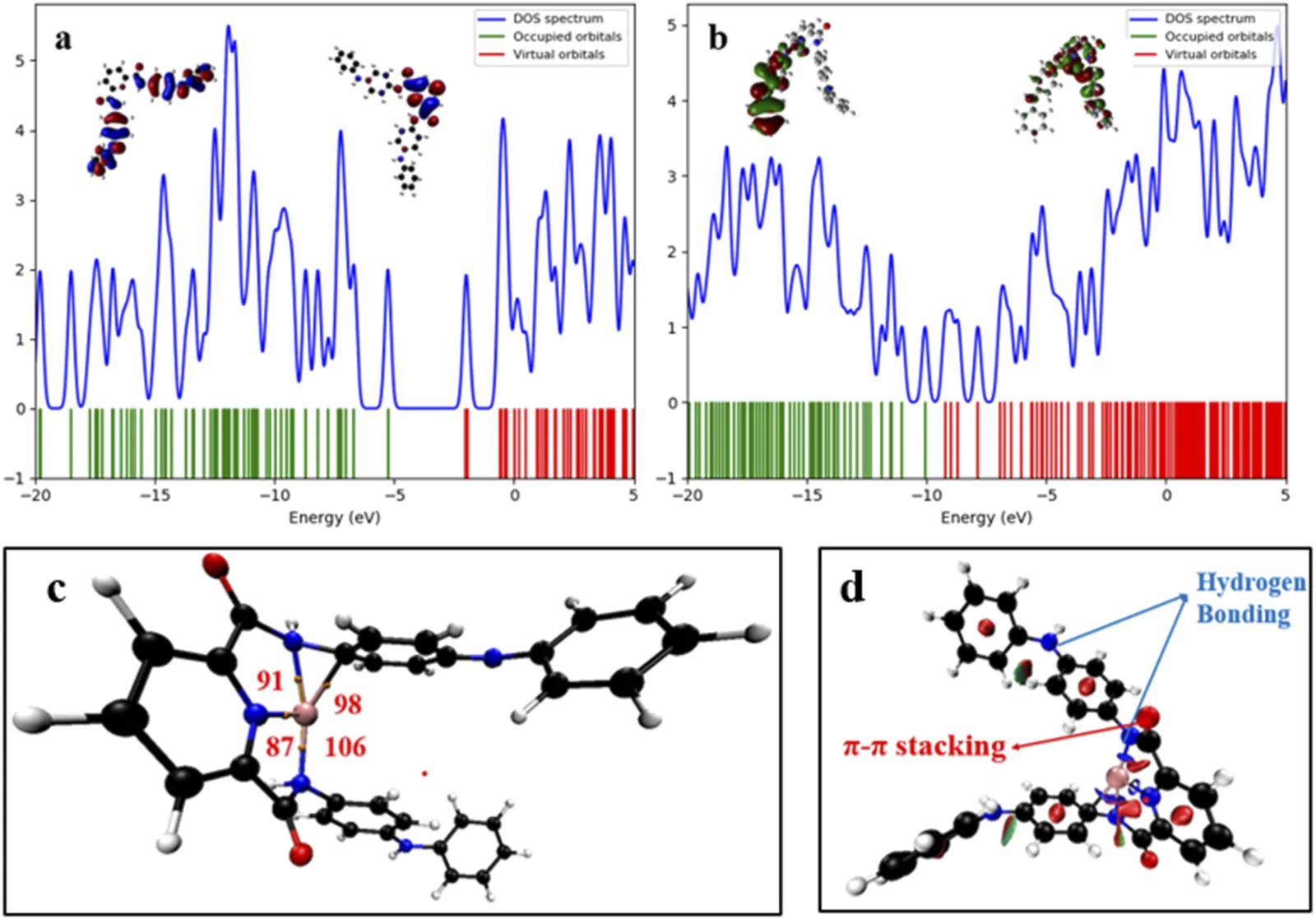
Density of states (DOS) spectra and frontier molecular orbital distributions of free DPD (a) and the DPD@Fe^2+^ complex (b) obtained from DFT calculations. QTAIM bond critical point (BCP) analysis illustrating the Fe^2+^@DPD interactions (c). Non-covalent interaction (3d-NCI) analysis showing the intermolecular hydrogen-bonding and π–π stacking interactions in the DPD@Fe^2+^ complex (d).

Overall, the combined FMO and DOS results confirm that Fe incorporation significantly modulates the electronic structure of DPD, producing a system with enhanced charge transfer capability and improved sensing performance.

#### QTAIM and NCI analysis

To investigate the non-covalent interactions between DPD and Fe^2+^, NCI analysis was performed using the Multiwfn program. The 3D-NCI isosurface map of the DPD@Fe^2+^ complex revealed the presence of attractive, dispersive, and steric interaction regions within the coordination framework. The prominent blue-colored isosurfaces around the Fe center indicate strong Fe-ligand attractive interactions formed through coordination with three nitrogen donor atoms and one carbon donor site, resulting in enhanced electron delocalization and stabilization of the complex (Fig. 7d). The green-colored regions correspond to weak van der Waals and dispersive interactions, whereas the red-colored isosurfaces represent steric repulsion arising from close atomic contacts around the coordination environment.

To further characterize the binding interactions, QTAIM analysis was carried out to identify the bond critical points (BCPs) involved in the DPD@Fe^2+^ complex. Four significant BCPs were observed, including N⋯Fe^2+^ (BCP 87), N⋯Fe^2+^ (BCP 91), Fe^2+^⋯N (BCP 98), and Fe^2+^⋯C (BCP 106) (Fig. 7c). The corresponding interaction energies were calculated to be −83.44, −144.67, −89.20, and −556.69 kcal mol^−1^, respectively, confirming the strong stabilization of the coordination framework (Table S3).

Moreover, the positive values of ∇^2^*ρ*(*r*) together with negative *H*(*r*) values suggest the partially covalent nature of the Fe-ligand interactions. Overall, the combined NCI and QTAIM analysis demonstrate that the DPD@Fe^2+^ complex possesses remarkable structural stability due to the synergistic contribution of coordination bonding, dispersive interactions, and steric effects.

### Applications

#### Rapid sensing in native biological substrates

To develop a practical solid-phase detection method, native potato was used as a natural source of iron (Fe^2+^) sensor substrate. Small potato pieces were soaked in a 2 mM solution of sensor DPD in DMF. Soaked potatoes were left undisturbed for 2 hours to allow the fluorophore to be immobilized within the natural matrix. A marked decrease in fluorescence intensity was observed in the treated potatoes when compared with untreated control (Fig. 8). This change in color is due to the direct interaction between the immobilized DPD molecules and Fe^2+^ ions naturally present in the potato tissue. This approach demonstrates a simple and real-time sensing strategy using an easily available biological material. The visible quenching response confirms that the DPD sensor remains functional in a solid, untreated substrate, providing a straightforward method for rapid solid-phase detection.

**Fig. 8:**
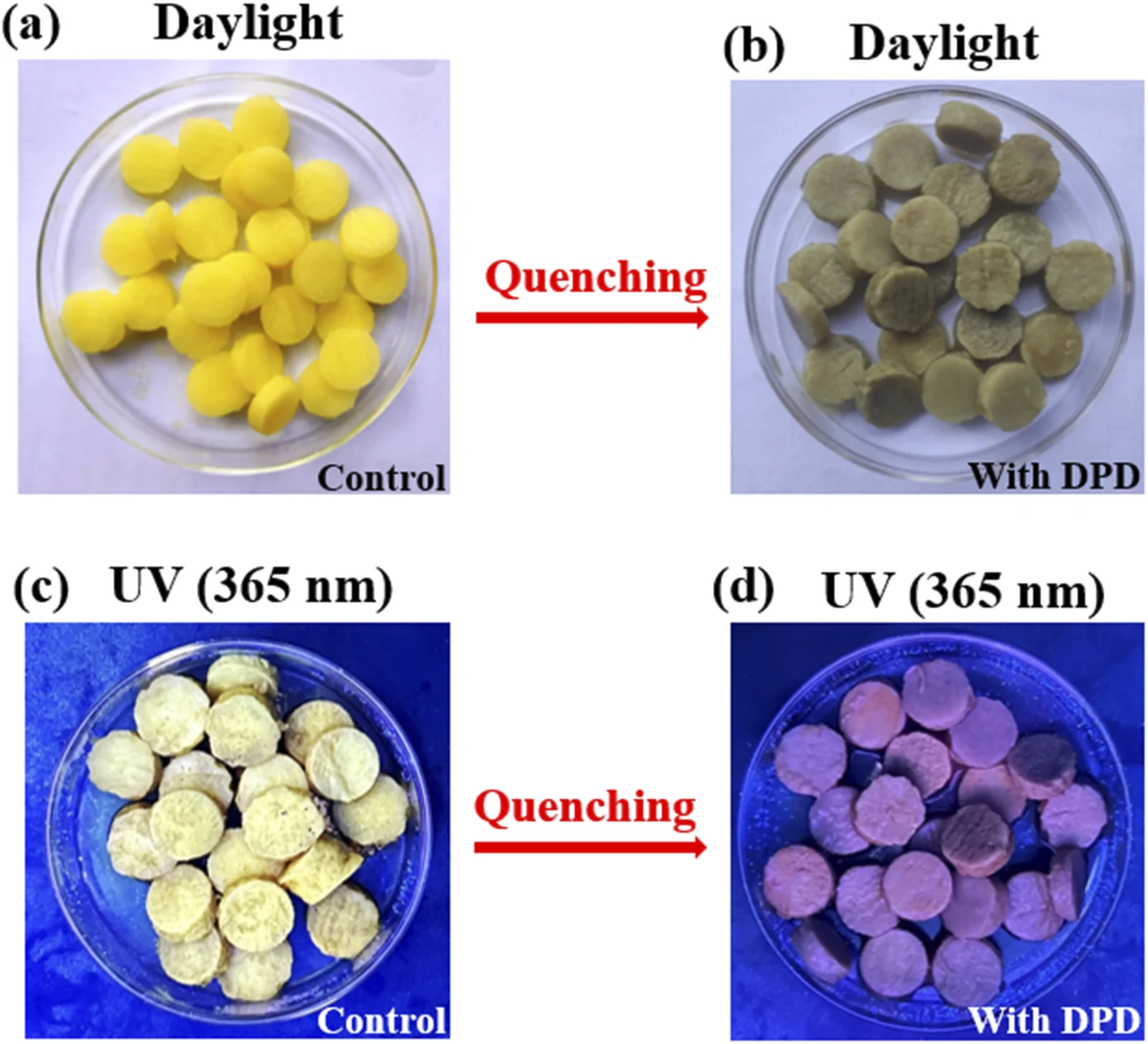
Solid phase detection of Fe^2+^ in native potato pieces using sensor DPD. Photographs recorded under daylight (a and b) and UV light at 365 nm (c and d). A noticeable decrease in fluorescence intensity is observed after interaction with Fe^2+^.

### Colorimetric and solid phase detection

To investigate the practical applicability of DPD for solid-phase Fe^2+^ detection, a TLC plate was uniformly coated with a 1.0 mM solution of the sensor DPD and allowed to dry. FeSO_4_ solution (1 mM) was then applied dropwise onto the coated surface using a dropper. The treated regions showed a distinct visual and fluorescence response compared with the untreated areas under daylight and 365 nm UV irradiation, respectively (Fig. 9). These observations confirm that DPD can detect Fe^2+^ on a solid support and demonstrate its potential for simple, rapid, and portable solid-phase sensing applications. For colorimetric sensing, 1 mM solutions of DPD and FeSO_4_ were prepared and transferred into glass vials. DPD exhibited a yellowish color under daylight and UV irradiation at 254 nm. Upon the addition of FeSO_4_, the color changed immediately to orange under daylight and brown under UV irradiation at 254 nm. These observations demonstrate the potential of DPD for the colorimetric detection of Fe^2+^ ions.

**Fig. 9:**
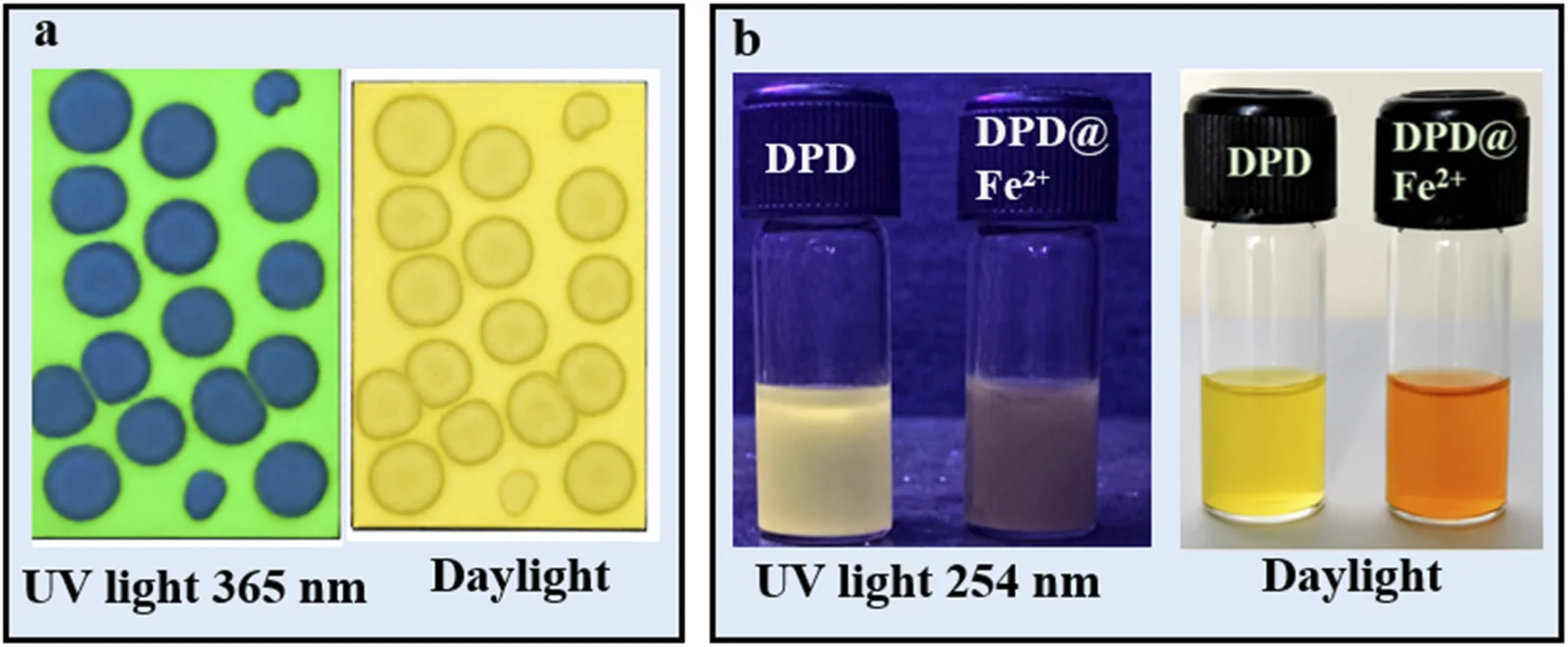
Solid-phase sensing on a DPD-coated TLC plate under 254 nm UV light and daylight (a). Colorimetric and fluorescence response of DPD after the addition of FeSO_4_ solution under daylight and 254 nm UV light (b).

### Colorimetric detection of acid in vapor phase

Sensor DPD was studied as a solid-state sensor for rapid detection of acidic vapors. A sensor strip was easily prepared by coating 1 mM solution of sensor DPD on a TLC plate. Upon exposure to trifluoroacetic acid (TFA) vapors, the strip showed an immediate and clear change in color under daylight and 365 nm UV light (Fig. 10).

**Fig. 10:**
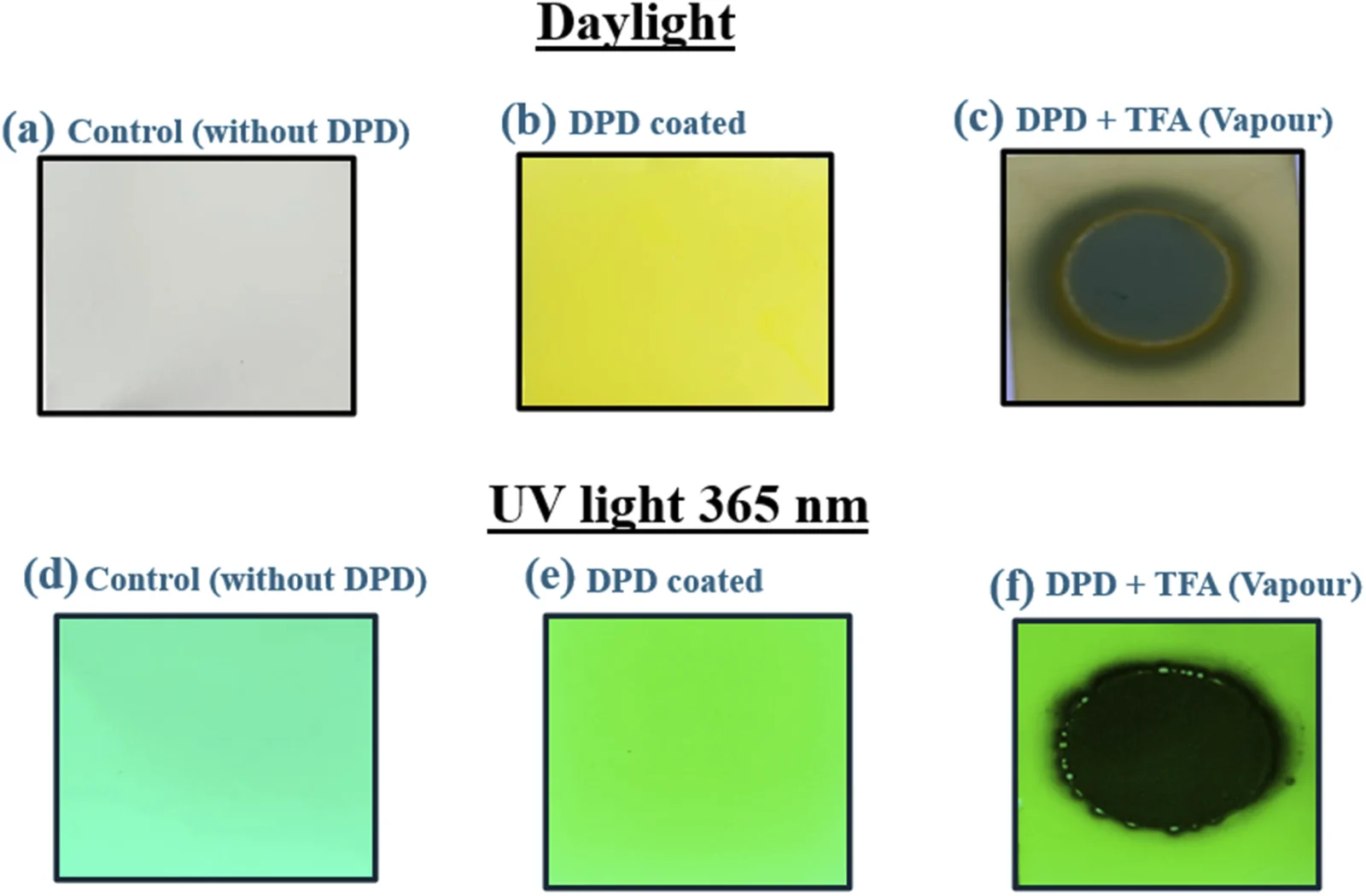
Colorimetric response of DPD-coated TLC plate for acid sensing under daylight and UV light (365 nm). (a and d) Control TLC plate without DPD, (b and e) DPD-coated TLC plate, and (c and f) DPD-coated TLC plate after exposure to acid vapors, showing a distinct visible and fluorescence color change.

This rapid colorimetric response highlights the potential of the sensor for detecting acidic species through visible color changes, offering a simple and convenient approach for on-site monitoring of acidic vapors without requiring sophisticated equipment.

## Conclusion

Fluorescent sensor DPD was successfully synthesized and characterized through various spectroscopic techniques. The sensor exhibited aggregation-induced emission enhancement in a DMF/H_2_O (1/9 v/v) system, demonstrating its strong emissive behavior in the aggregated state. DPD was successfully employed for the selective detection of Fe^2+^ ions through a pronounced fluorescence quenching response, achieving high quenching efficiency. The sensing mechanism and interaction between DPD and Fe^2+^ were validated by FTIR, ^1^H NMR titration, cyclic voltammetry and LC-MS titration analysis. The selectivity of DPD toward Fe^2+^ was further confirmed through interference studies in the presence of various competing analytes and interfering species. Furthermore, investigations of response time, photostability, temperature, and pH demonstrated the rapid response and good stability of the sensor under different experimental conditions. Theoretical studies based on frontier molecular orbital analysis highlighted the electronic changes responsible for fluorescence quenching upon Fe^2+^ interaction. In addition, DPD showed promising practical applicability through visible colorimetric detection and solid-phase sensing of Fe^2+^ using sensor-coated TLC plates. These findings indicate that DPD is a simple, selective, stable, and practical fluorescent platform for the detection of Fe^2+^ ions in analytical and environmental applications.

## Author contributions

Bushra Jadoon: methodology, investigation, writing – original draft. Alam Shabbir: methodology, formal analysis, writing – original draft. Asad Muhammad Khan: methodology, investigation. Mohammed A. Assiri: methodology, visualization, software, formal analysis, funding acquisition. Umar Farooq: investigation, supervision. Sohail Anjum Shahzad: conceptualization, methodology, investigation, validation, supervision, project administration, funding acquisition, writing – review & editing.

## Conflicts of interest

The authors declare that there are no conflicts to declare.

## Supplementary Material

RA-OLF-D6RA06979G-s001

RA-OLF-D6RA06979G-s002

RA-OLF-D6RA06979G-s003

## Data Availability

The supporting data has been provided as a part of the supplementary information (SI). Supplementary information: comparison of reported fluorescent probes, theoretical studies, DLS, NMR, and LC-MS titrations, overlay FTIR spectra of free DPD and complex (DPD@Fe^2+^), cyclic voltammograms, photophysical studies *e.g.*: SV plots, pH, temperature effects, photostability, and selectivity studies in figures and tabular forms. See DOI: https://doi.org/10.1039/d6ra06979g.
