# Supplementary material for: Aggregation induced emission based fluorescent and colorimetric sensor for selective detection of Fe2+: experimental and computational insights with real-time applications
Source: RSC Adv. 2026 Nov 9. Online ahead of print. doi: 10.1039/d6ra06979g (PMC13746912; doi:10.1039/d6ra06979g)
Supplement: Supplementary file 1 [file RA-OLF-D6RA06979G-s001.pdf]

Supplementary Information (SI) for RSC Advances.
This journal is © The Royal Society of Chemistry

Supporting Information

Aggregation Induced Emission Based Fluorescent and Colorimetric Sensor for Selective Detection of Fe^{2+}: Experimental and Computational Insights with Real-Time Applications

Bushra Jadoon[a], Alam Shabbir[a], Asad Muhammad Khan[a], Mohammed A. Assiri[b,c], Umar Farooq[a,*], Sohail Anjum Shahzad[a,*]

[a] *Department of Chemistry, COMSATS University Islamabad, Abbottabad Campus, University Road, Abbottabad 22060, Pakistan*

[b] *Department of Chemistry, Faculty of Science, King Khalid University, P.O. Box 9004, Abha 61413, Saudi Arabia*

[c] *Central Labs, King Khalid University, AlQura'a, Abha, P.O. Box 960, 61413, Saudi Arabia*

* Corresponding authors: Sohail Anjum Shahzad, Department of Chemistry, COMSATS University Islamabad, Abbottabad Campus, University Road, Abbottabad 22060, Pakistan.

E-mail addresses: umarf@cuiatd.edu.pk (U. Farooq); sashahzad@cuiatd.edu.pk (S. A. Shahzad)

Supplementary Material

The fluorescence measurements of sensor DPD were executed by spectrofluorometer ", spectrofluorometer Perkin Elmir LS 45 while UV-Vis. absorption spectra was recorded at UV Vis Spectrophotometer Double Beam Shimadzu 1601, Japan. Bruker Avance III HD 400 MHz NMR spectrometer was used for the structure characterization and titration experimentations. All chemicals and reagents were purchased from Daejung Chemicals & Metals (Korea), Alfa Aesar (UK), and Sigma Aldrich (USA), and utilized without purification. Reagents include N,N-dimethylformamide (DMF), methanol (MeOH), tetrahydrofuran (THF), dimethyl sulfoxide (DMSO), dichloromethane (DCM), chloroform ($CHCl_3$), and acetonitrile (ACN). Analytes include Barium chloride ($BaCl_2$), Magnesium sulfate ($MgSO_4$), Sodium nitrate ($NaNO_3$), Calcium chloride ($CaCl_2$), Sodium fluoride (NaF), Iron(III) nitrate [$Fe(NO_3)_3$], Nickel(II) nitrate [$Ni(NO_3)_2$], Sodium cyanide (NaCN), Zinc nitrate ($Zn(NO_3)_2$), Iron(II) sulfate ($FeSO_4$), Lithium hydroxide (LiOH), Manganese(II) sulfate ($MnSO_4$), Potassium cyanide (KCN), and Chromium(III) sulfite [$Cr_2(SO_3)_3$].

Table S1: Comparison of reported fluorescent probes for Fe^{2+} detection, including sensing mechanisms, fluorescence responses, limits of detection, and selectivity.

Probe	Fluorescence mechanism	Response	Limit of detection	Selectivity	References
**DPD (our work)**	**PET**	**Turn off**	**4.10 µM**	**Fe^{2+}**	-
Chalcone-based probe 5	Complexation	Turn off	47.9 µM (4.79 $\times 10^{-5}$ M)	Fe^{2+}	1
Cyanoimidazopyridine (SS1)	RICT	Turn off	55 µM	Cu^{2+}, Fe^{3+}, Fe^{2+}	2
Imidazole-functionalized BODIPY (BODIPY-3)	Metal-ion-induced fluorescence quenching	Turn off	28.665 µM	Cu^{2+}, Fe^{3+}, Fe^{2+}	3
(Ni-MOF)	FRET	Turn off	25.03 µM	Fe^{2+} and Fe^{3+}	4
Pyrene-functionalized Schiff-base chitosan (Chit-Pyr)	PET	Turn off	2.52	Cu^{2+}, Fe^{3+}, Fe^{2+}	5
curcumin-immobilized zeolitic imidazolate framework-8.	Indicator Displacement Assay (IDA)	Turn off	7.64 µM	Fe^{2+} and Cu^{2+}	6
S–N-CDs/PVA film	fluorescence quenching through metal–oxygen coordination	Turn-off	8.84 µM	Fe^{2+}, Co^{2+}, Cu^{2+} and Ni^{2+}.	7

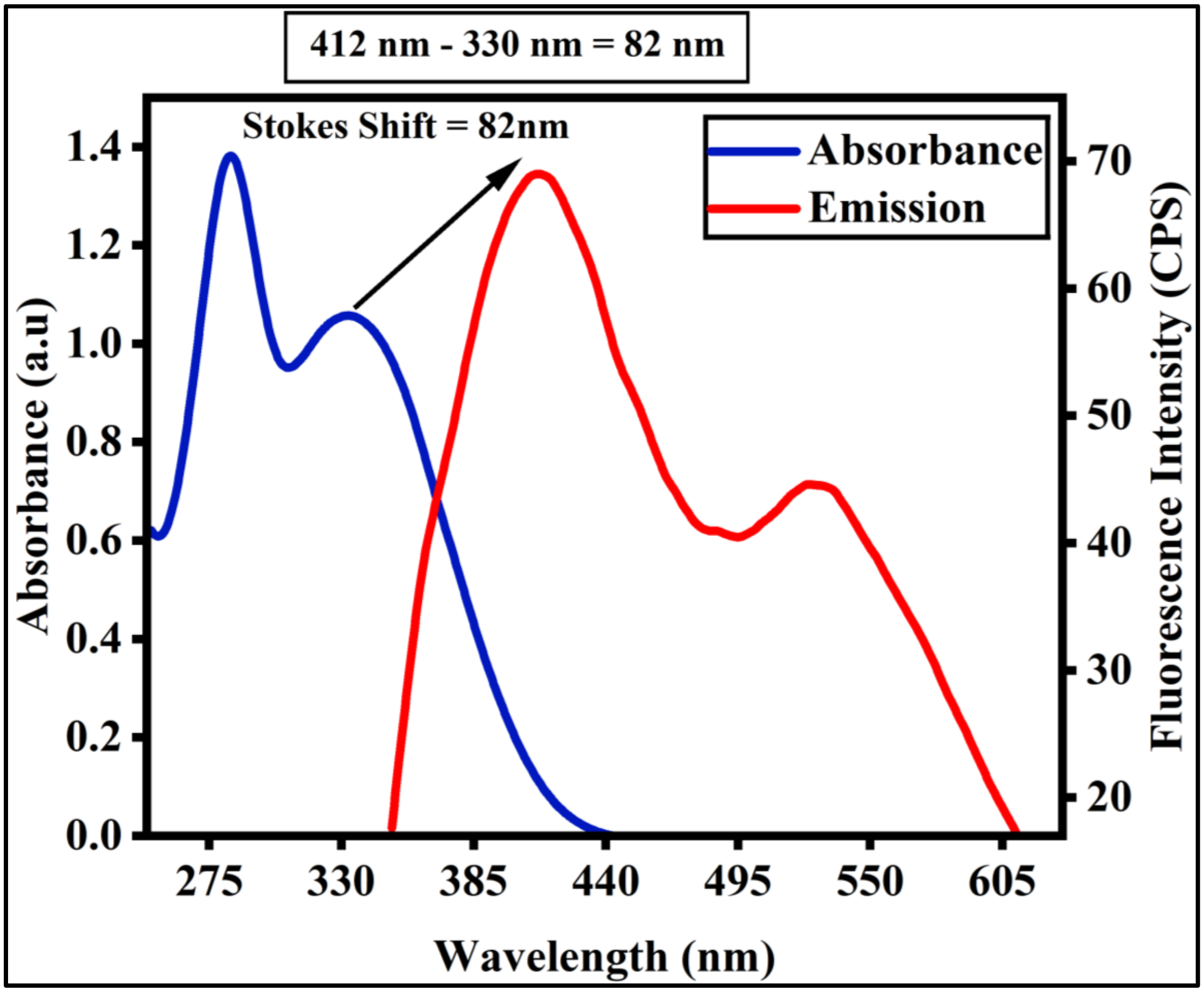

Figure S1: UV-Visible absorbance and emission of probe DPD in DMF solution (30 μM).

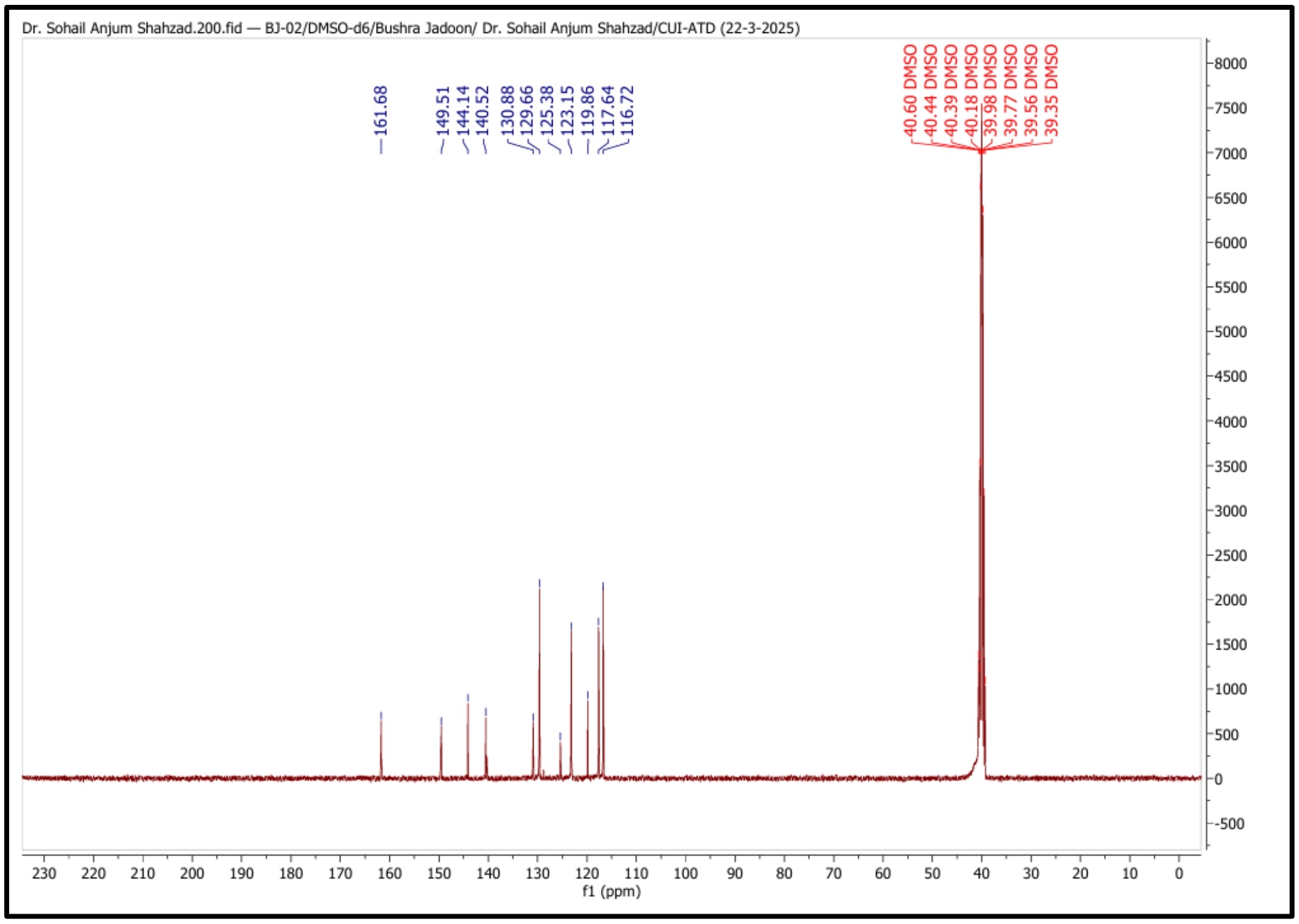

Figure S2: ^{13}C NMR spectrum of probe **DPD** recorded in DMSO-d$_6$.

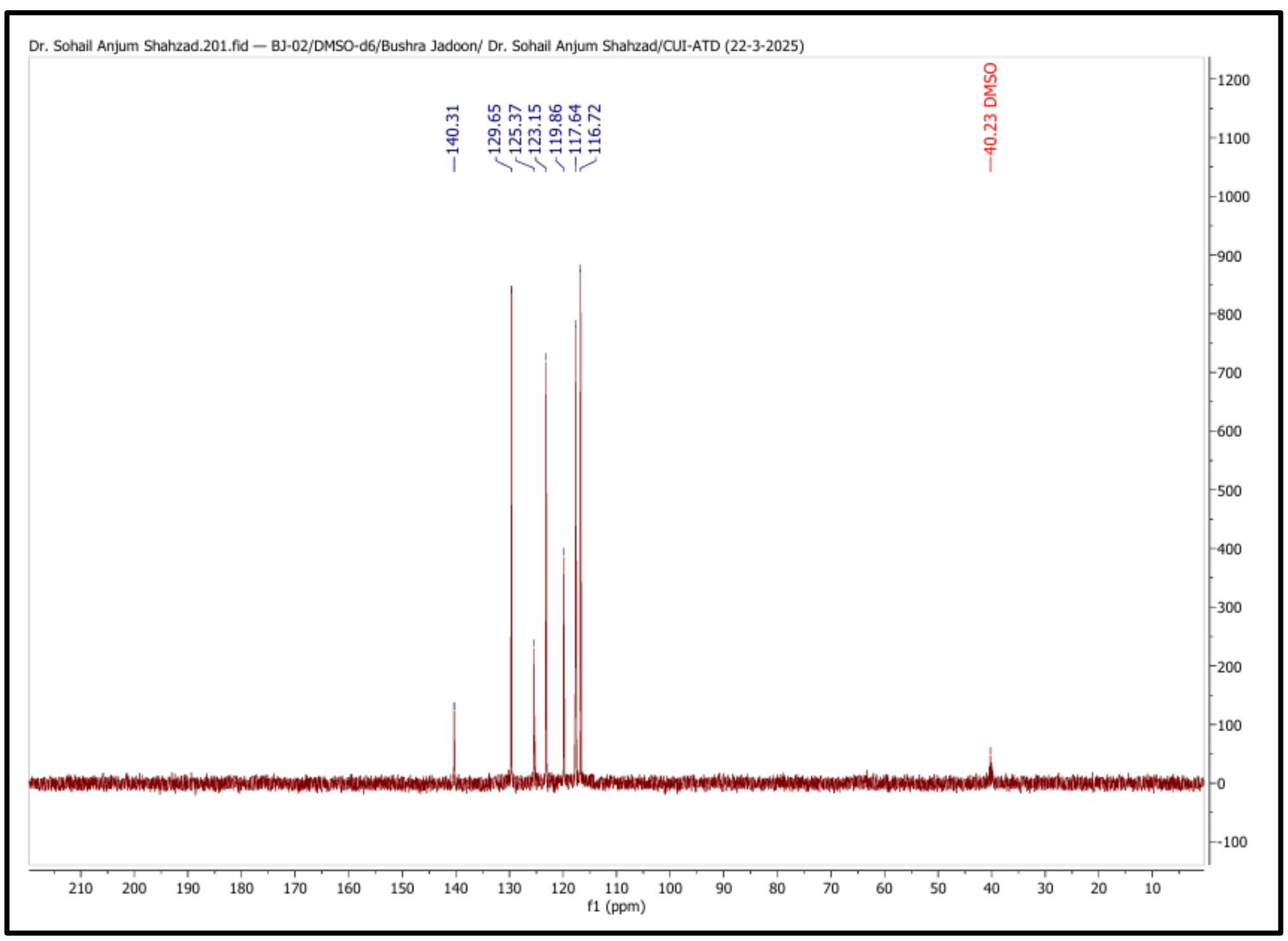

Figure S3: DEPT-135 NMR spectrum of probe **DPD** recorded in DMSO-d$_6$.

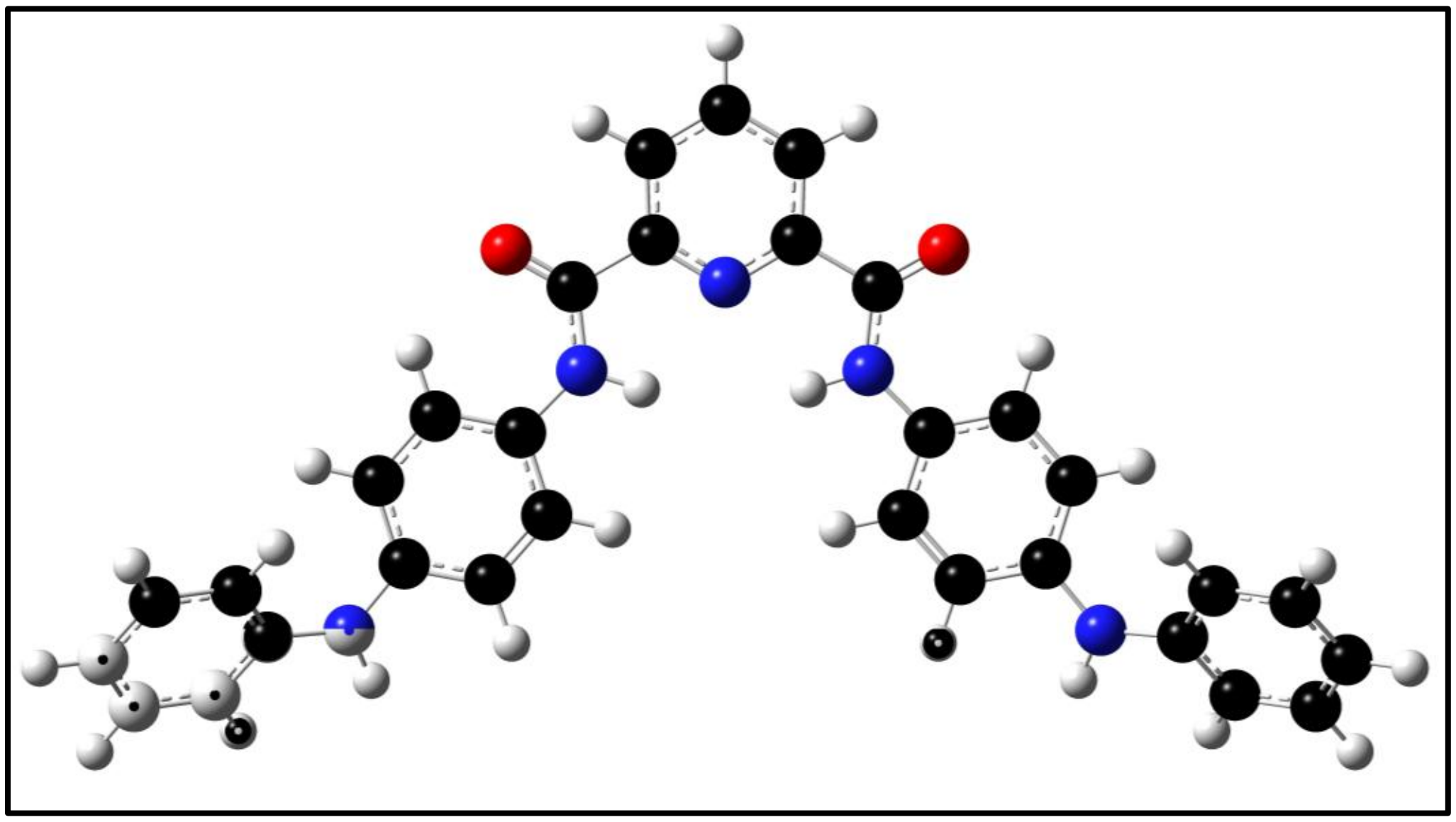

Figure S4: Optimized geometry of Probe **DPD** at B3LYP/6-311G

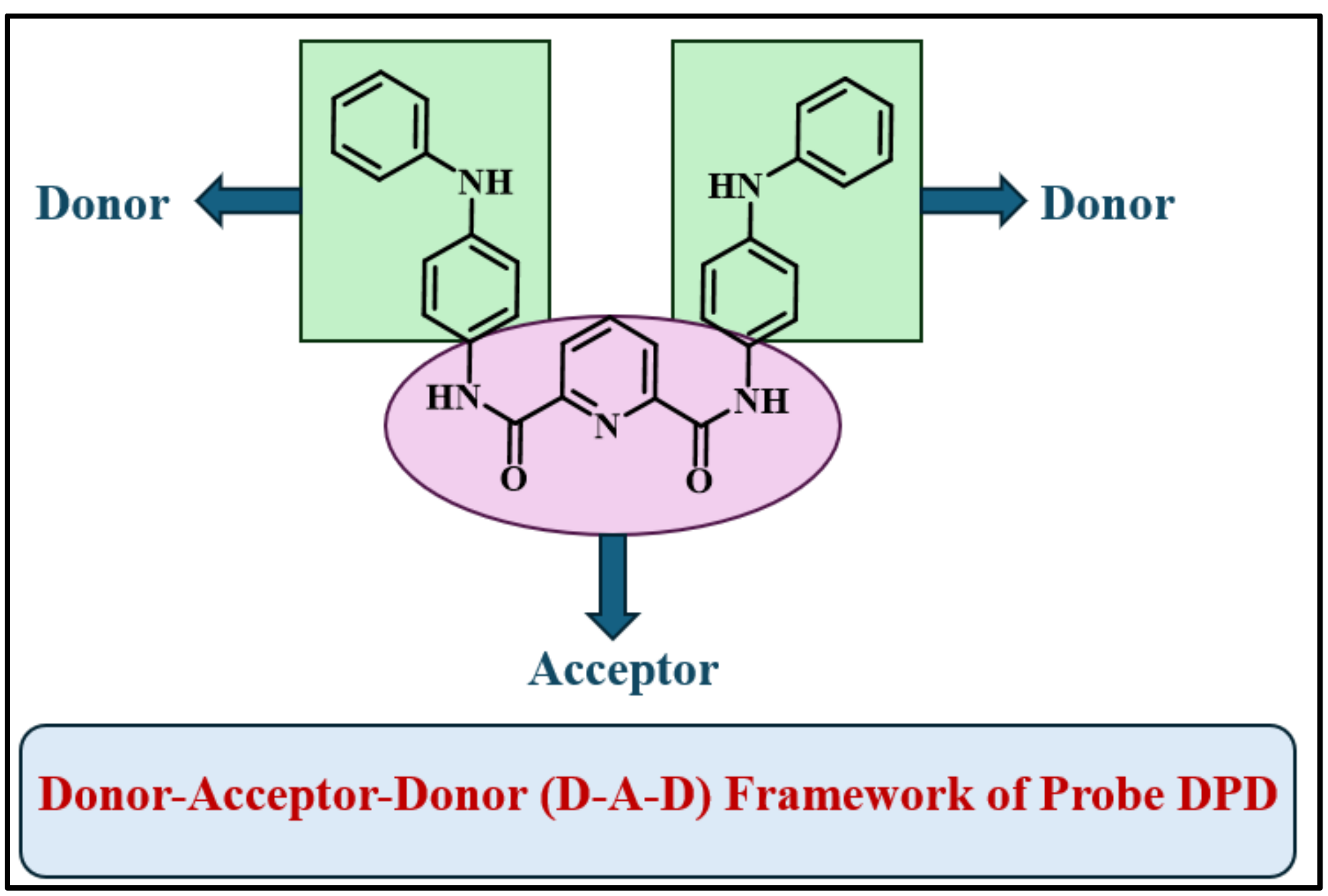

Figure S5: Chemistry of Probe **DPD** showing D-A-D framework.

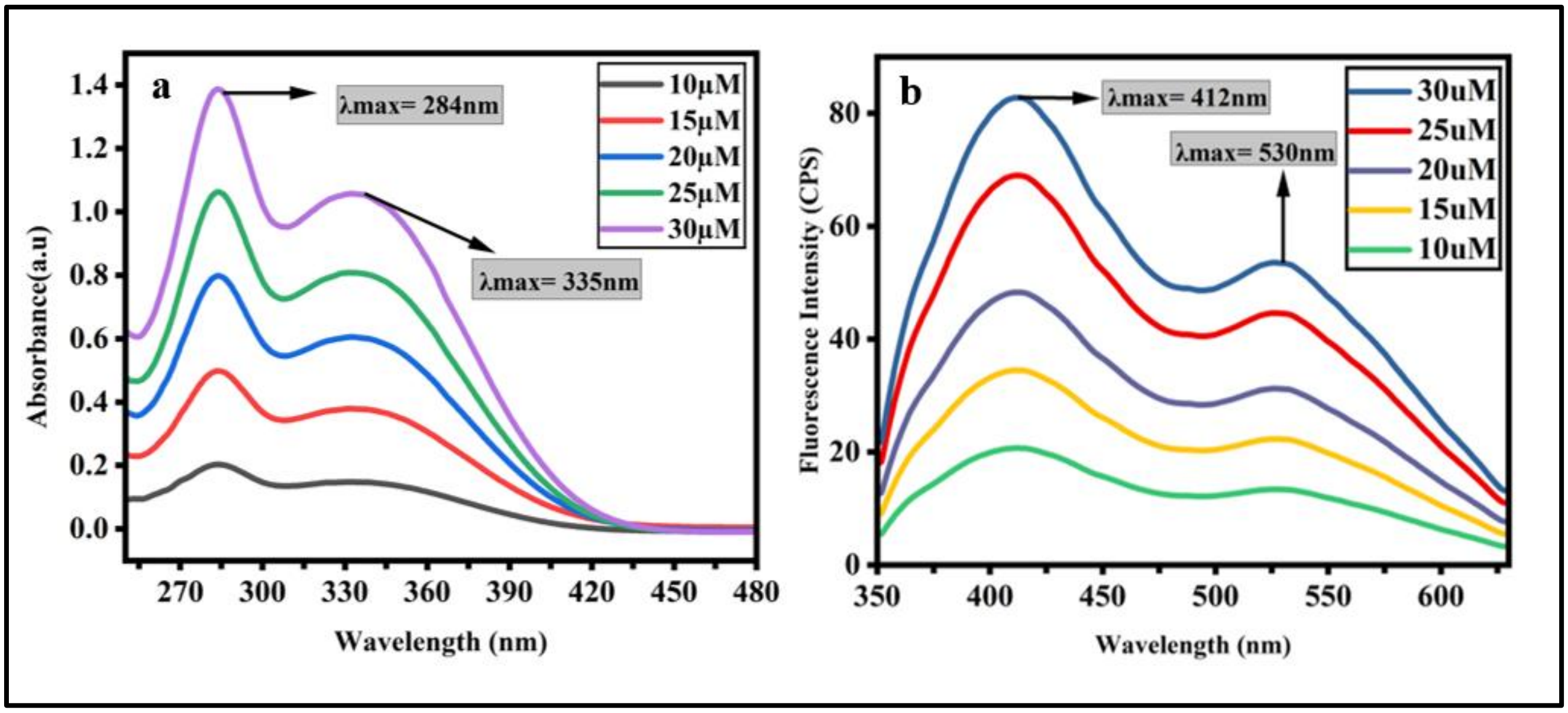

Figure S6: Optimization of the probe **DPD** concentration by UV-Vis absorption and fluorescence spectroscopy. (a) UV-Vis absorption spectra of the synthesized probe recorded at concentrations ranging from 10 to 30 μM. (b) Corresponding fluorescence emission spectra of the probe.

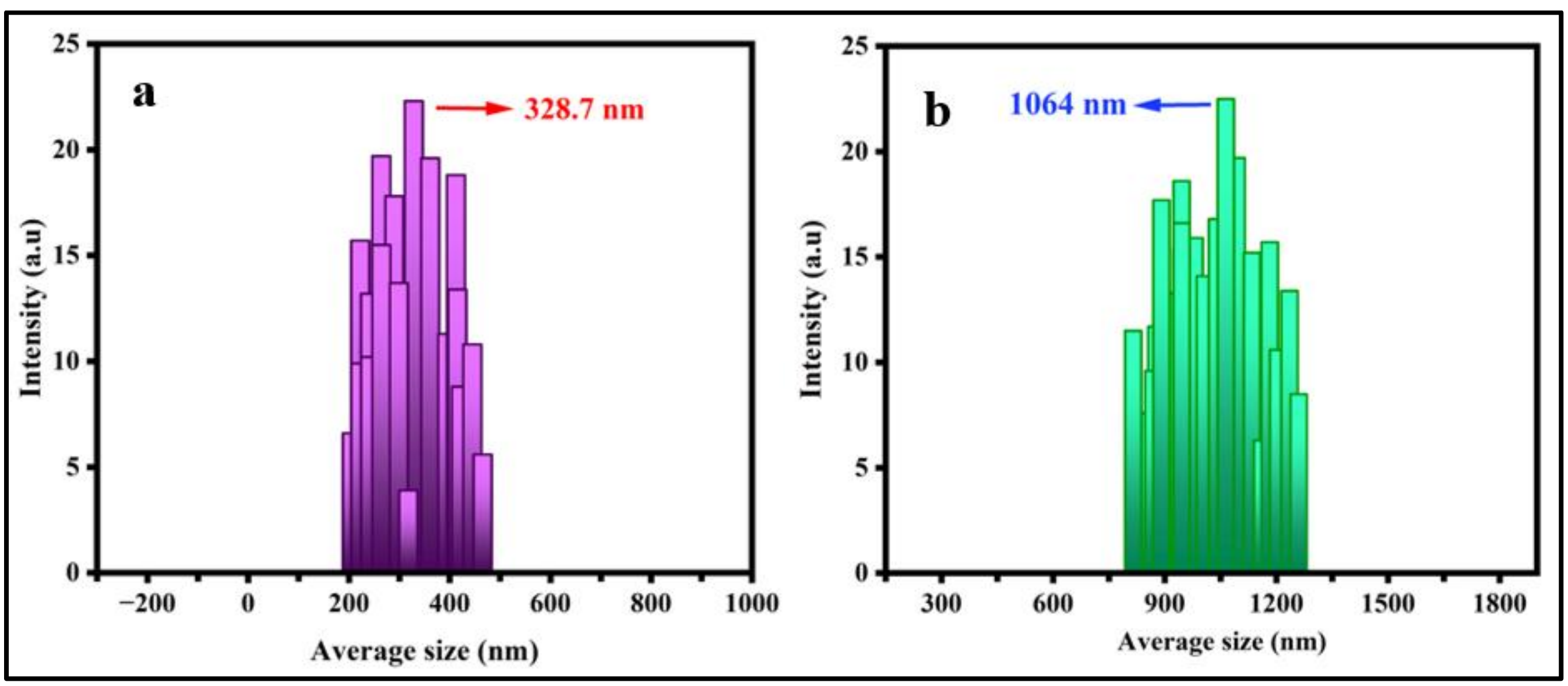

Figure S7: Dynamic light scattering (DLS) analysis of the synthesized probe **DPD**. Particle size distribution of the probe in 0% water fraction (a). Particle size distribution of the probe in 90% water fraction (b).

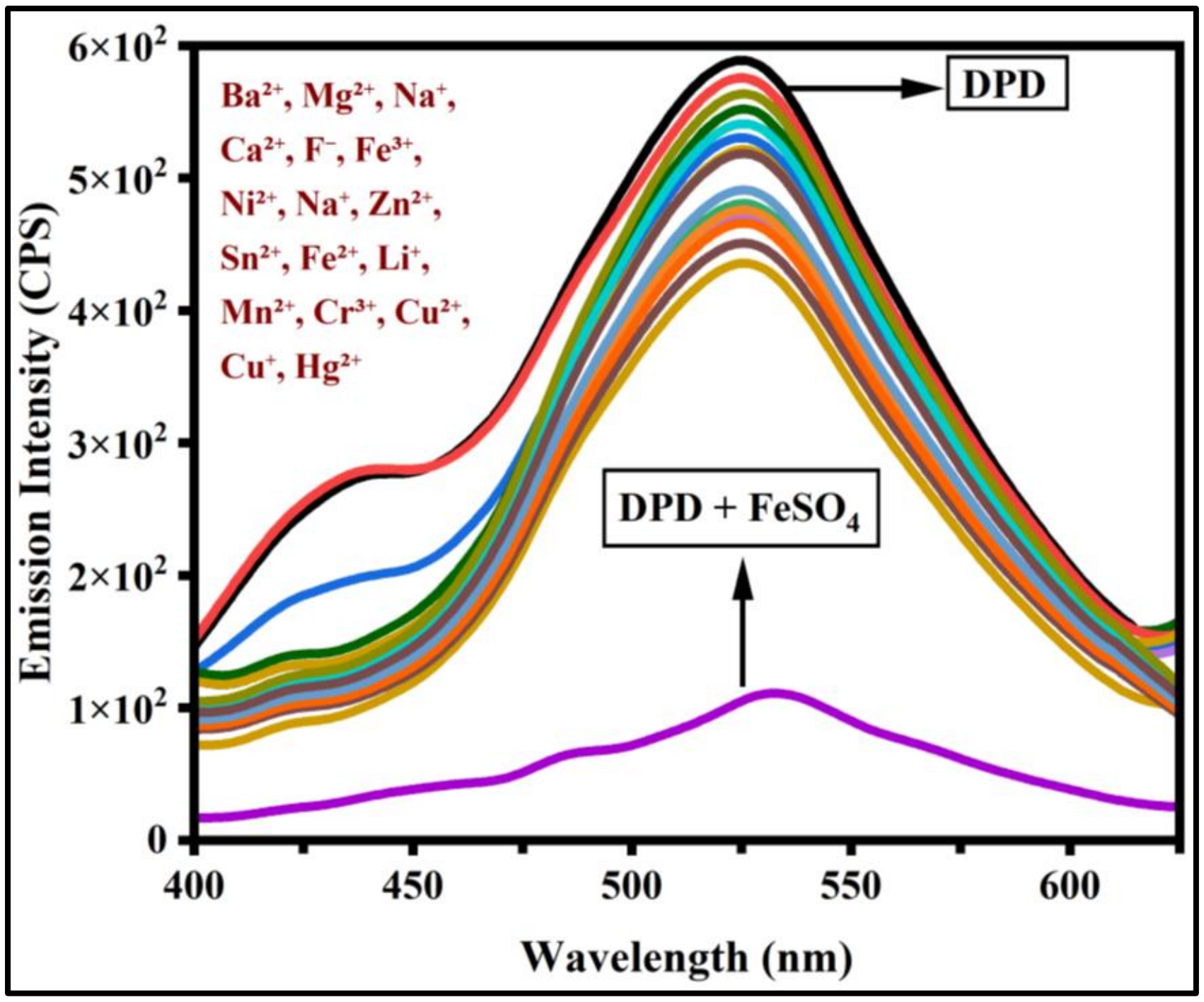

Figure S8: Selectivity of the **DPD** fluorescent probe toward Fe^{2+} over various metal ions, monitored by fluorescence emission spectroscopy.

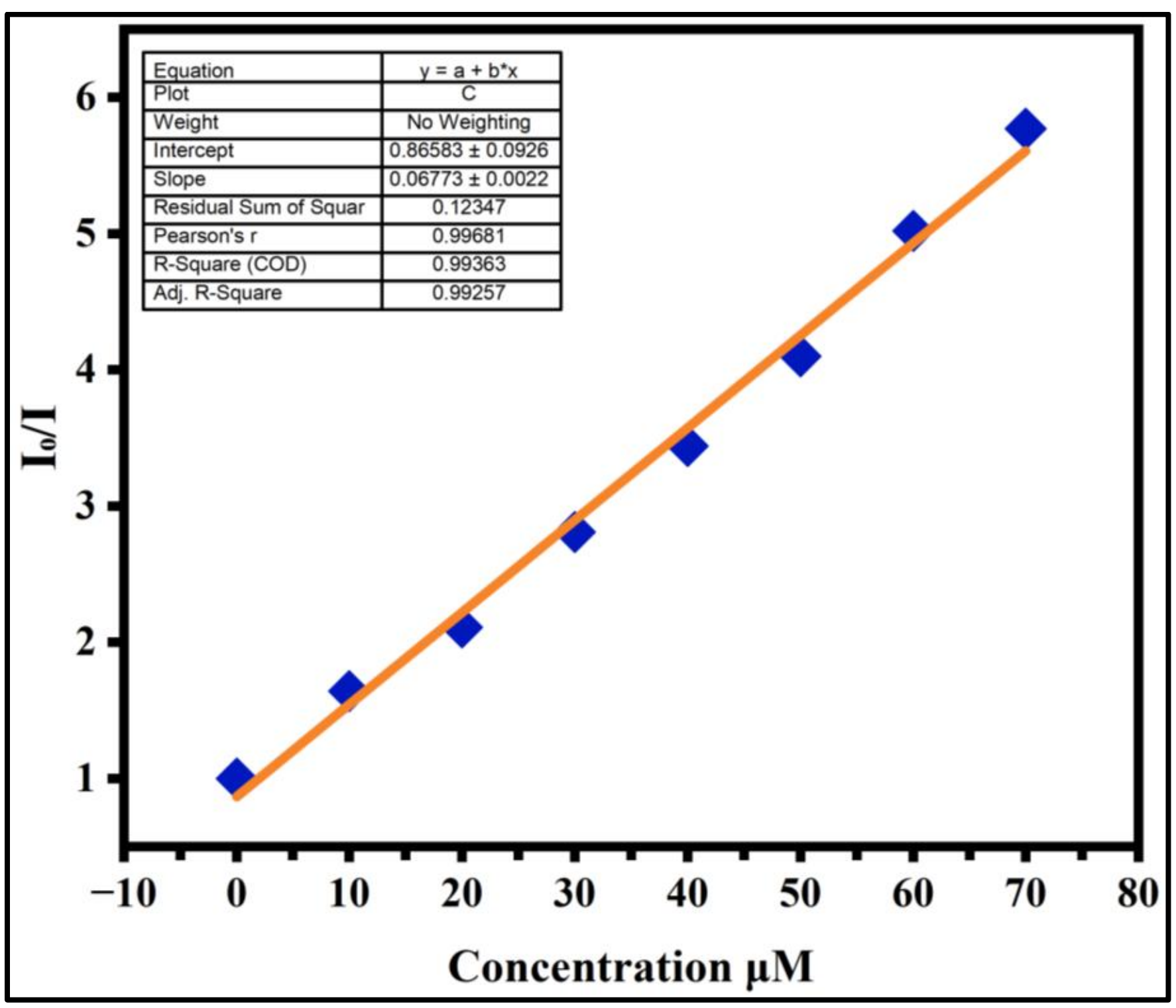

Figure S9: Stern-Volmer (SV) plot for Fe^{2+}induced fluorescence quenching of probe **DPD**

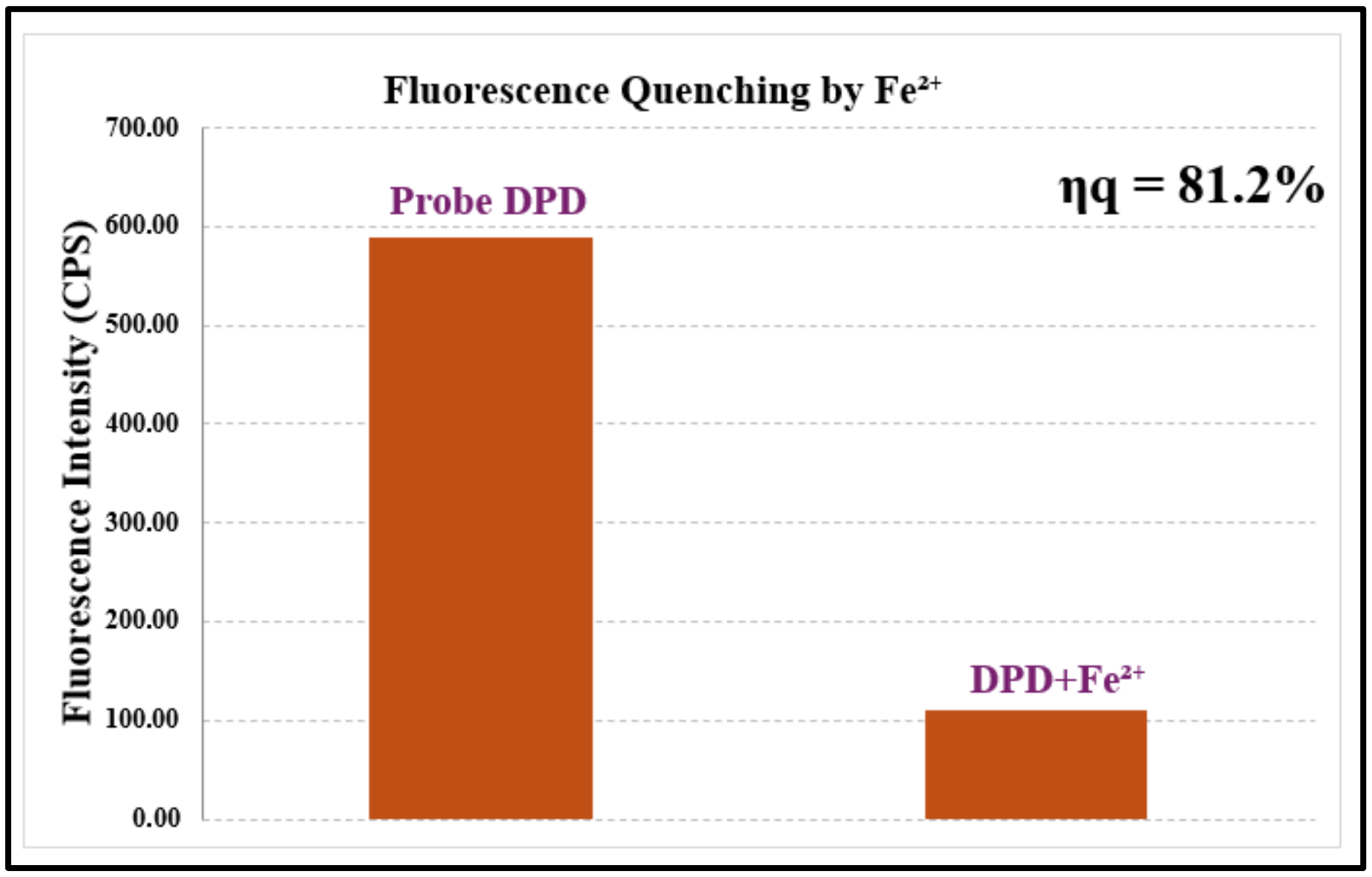

Figure S10: Fluorescence intensity of free **DPD** and **DPD@Fe^{2+}**, showing an 81.2% quenching efficiency upon addition of Fe^{2+}.

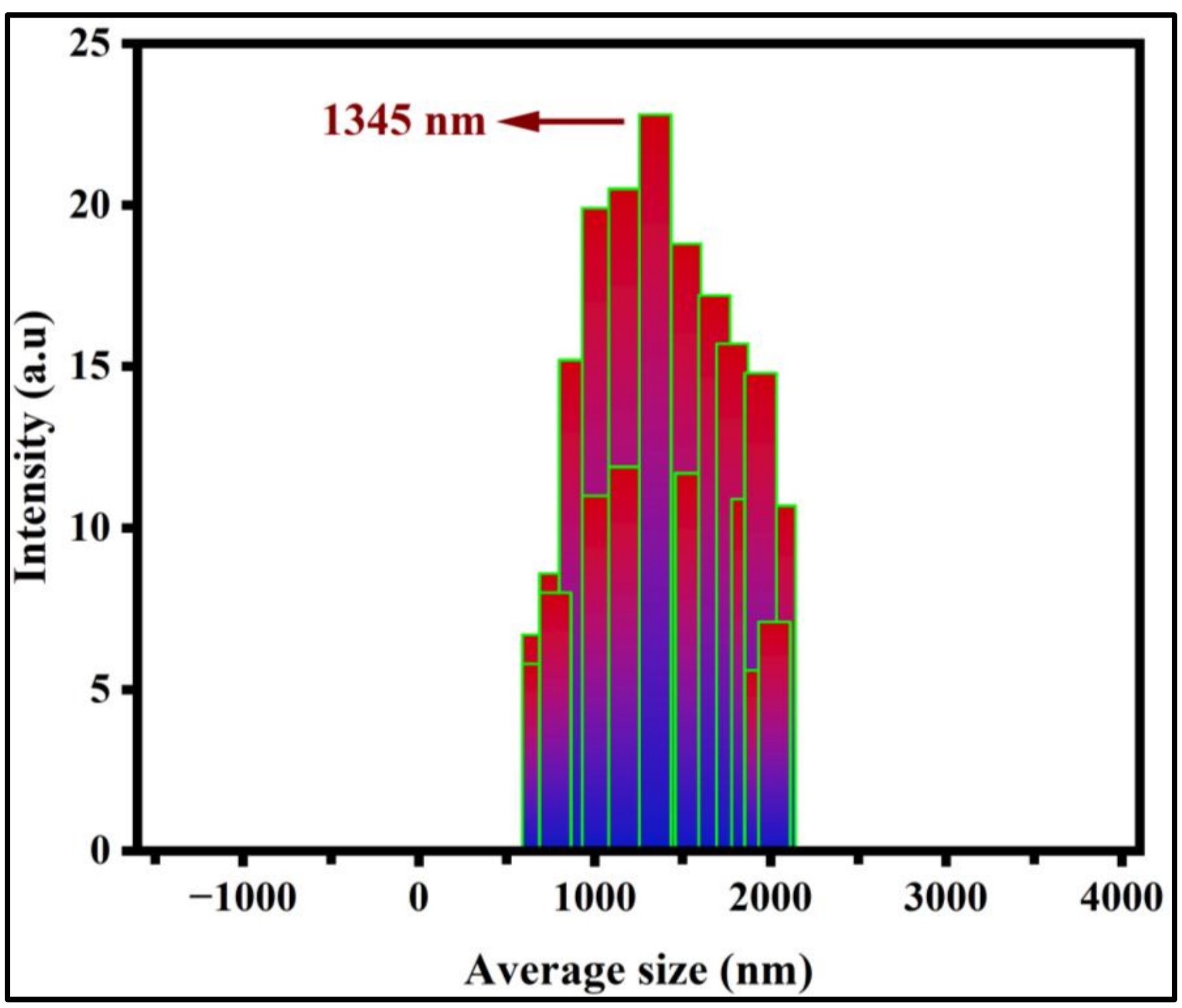

Figure S11: Dynamic light scattering (DLS) particle size distribution of the **DPD@Fe^{2+}** aggregates, showing an average hydrodynamic diameter of 1345 nm.

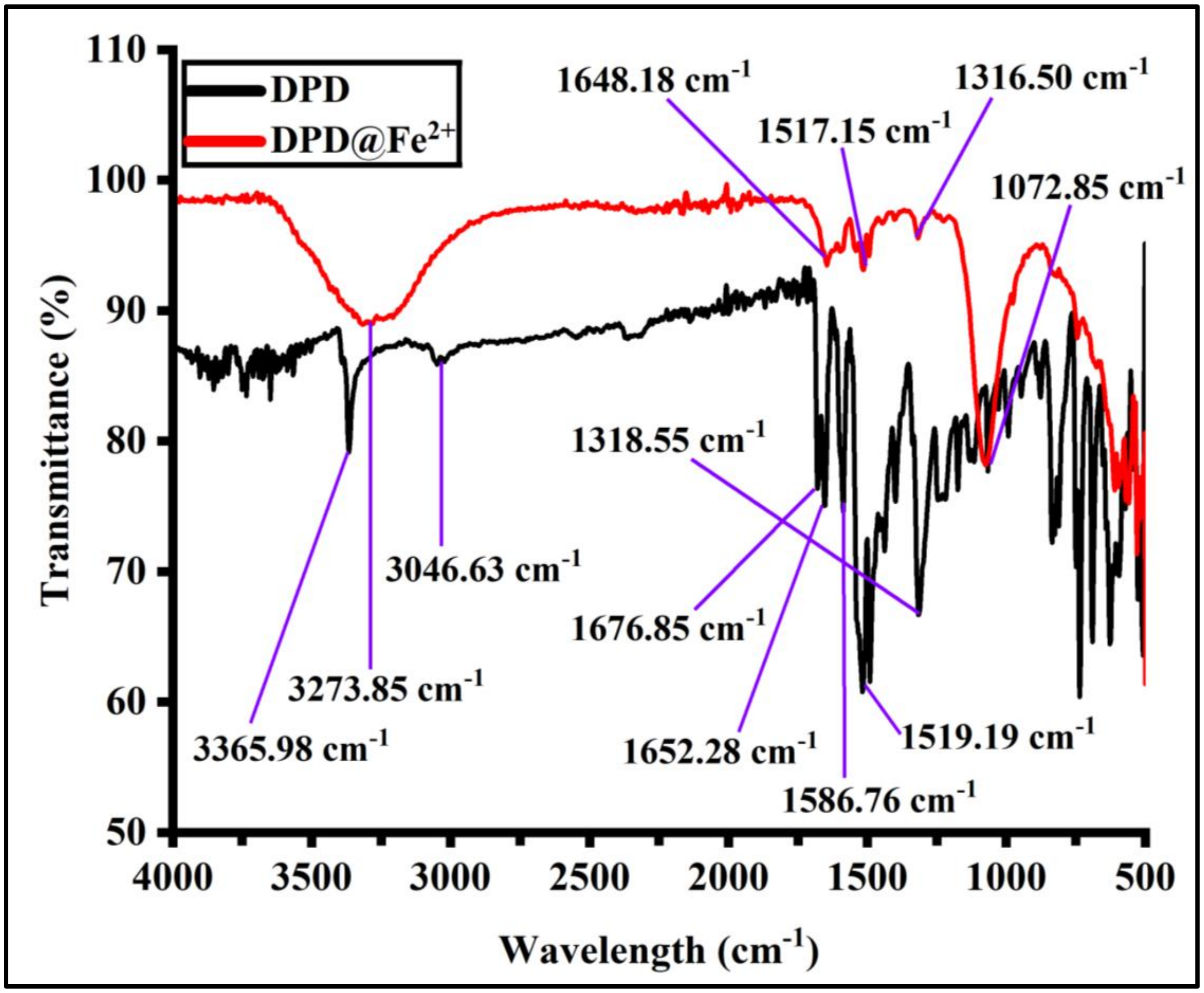

Figure S12: Overlay FTIR spectra of free **DPD** and complex (**DPD@Fe^{2+}**), showing characteristic band shifts upon Fe^{2+} interaction with DPD.

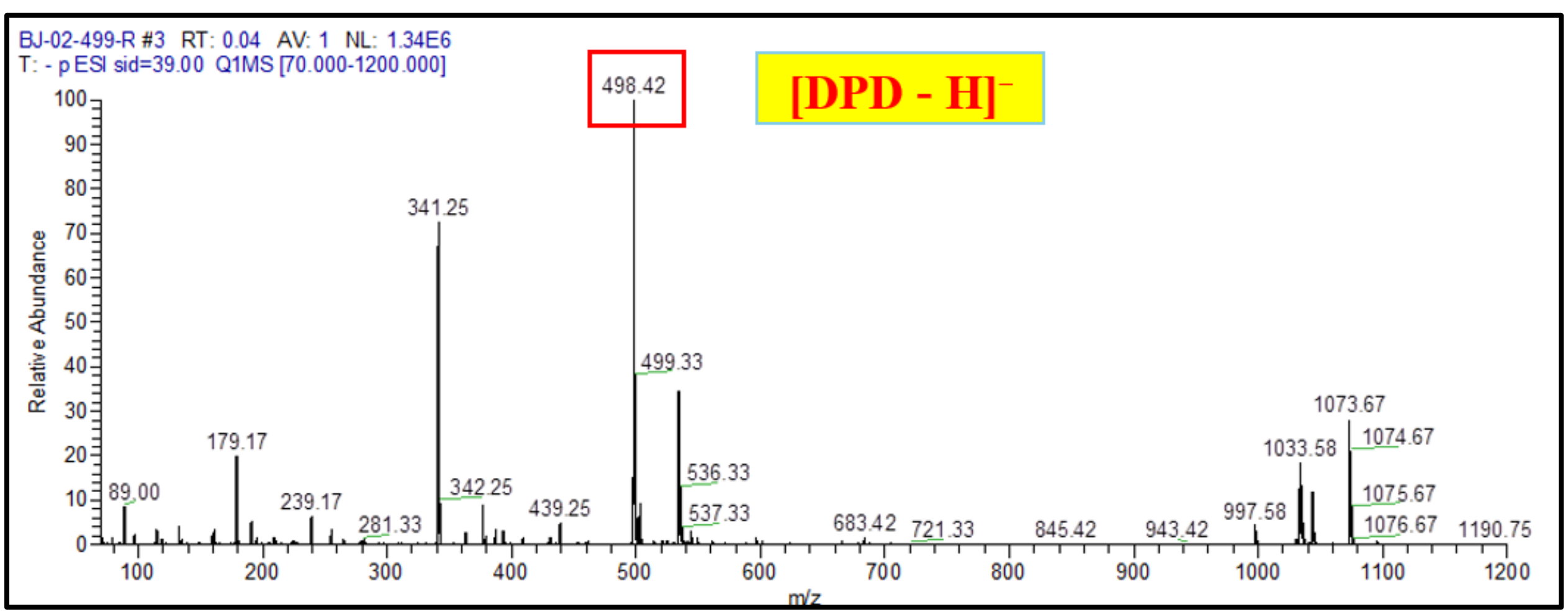

Figure S13: Negative mode ESI-MS spectrum of **DPD** showing the deprotonated molecular ion peak *at* m/z 498.42.

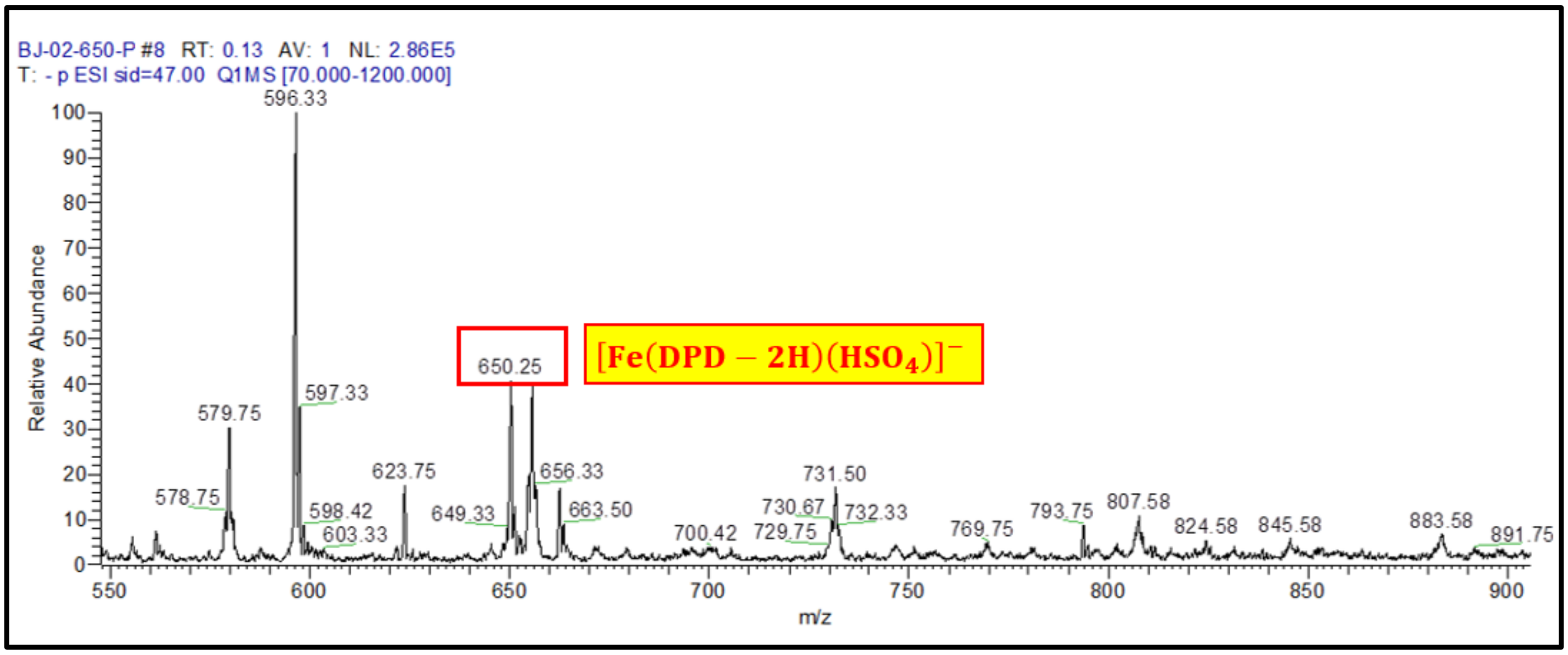

Figure S14: Negative-mode ESI-MS spectrum of the **DPD@Fe²⁺** complex showing the highlighted ion peak at m/z 650.25, tentatively assigned to $[Fe(DPD\text{-}2H)(HSO_4)]^-$.

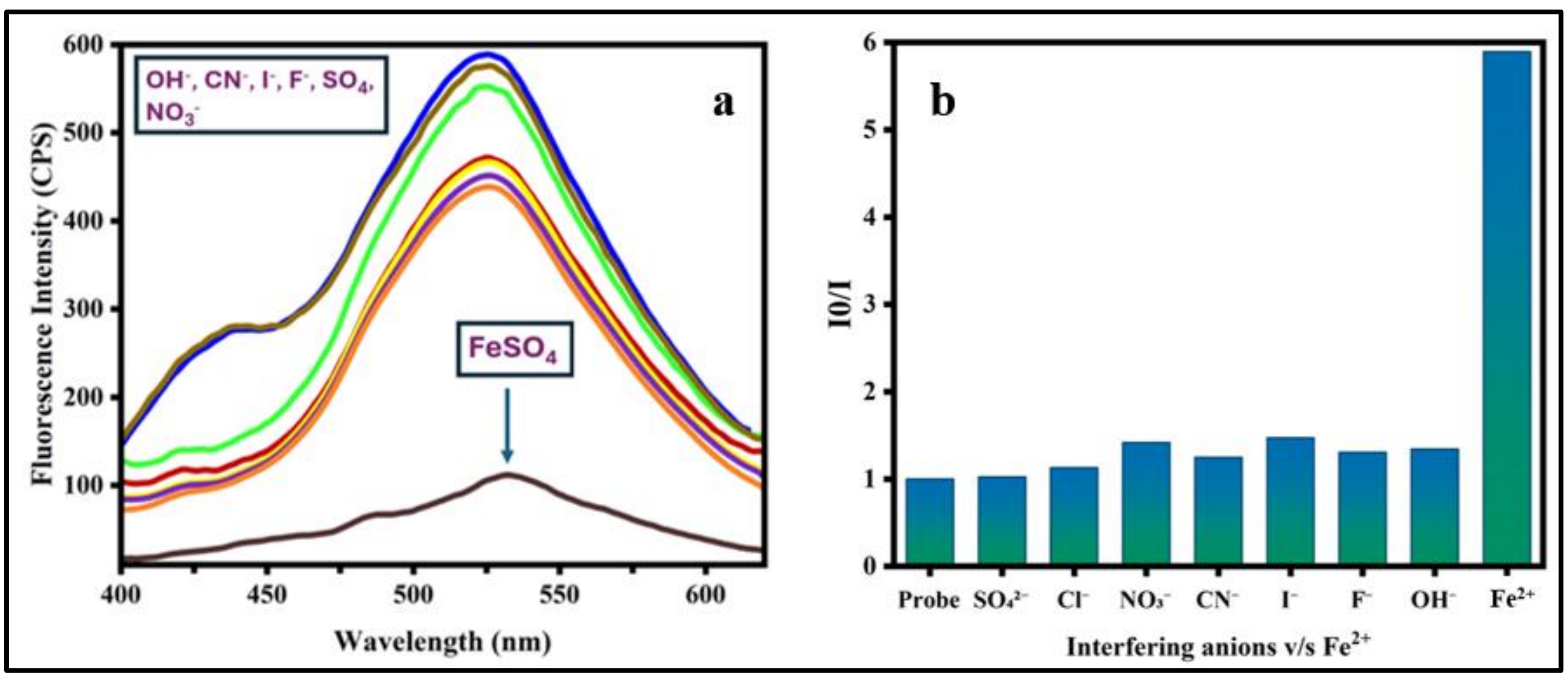

Figure S15: Fluorescence emission spectra of **DPD** in the presence of interfering anions and Fe^{2+}(a). Relative fluorescence response (I_0/I) of **DPD** toward interfering anions and Fe^{2+} (1.0 mM) (b).

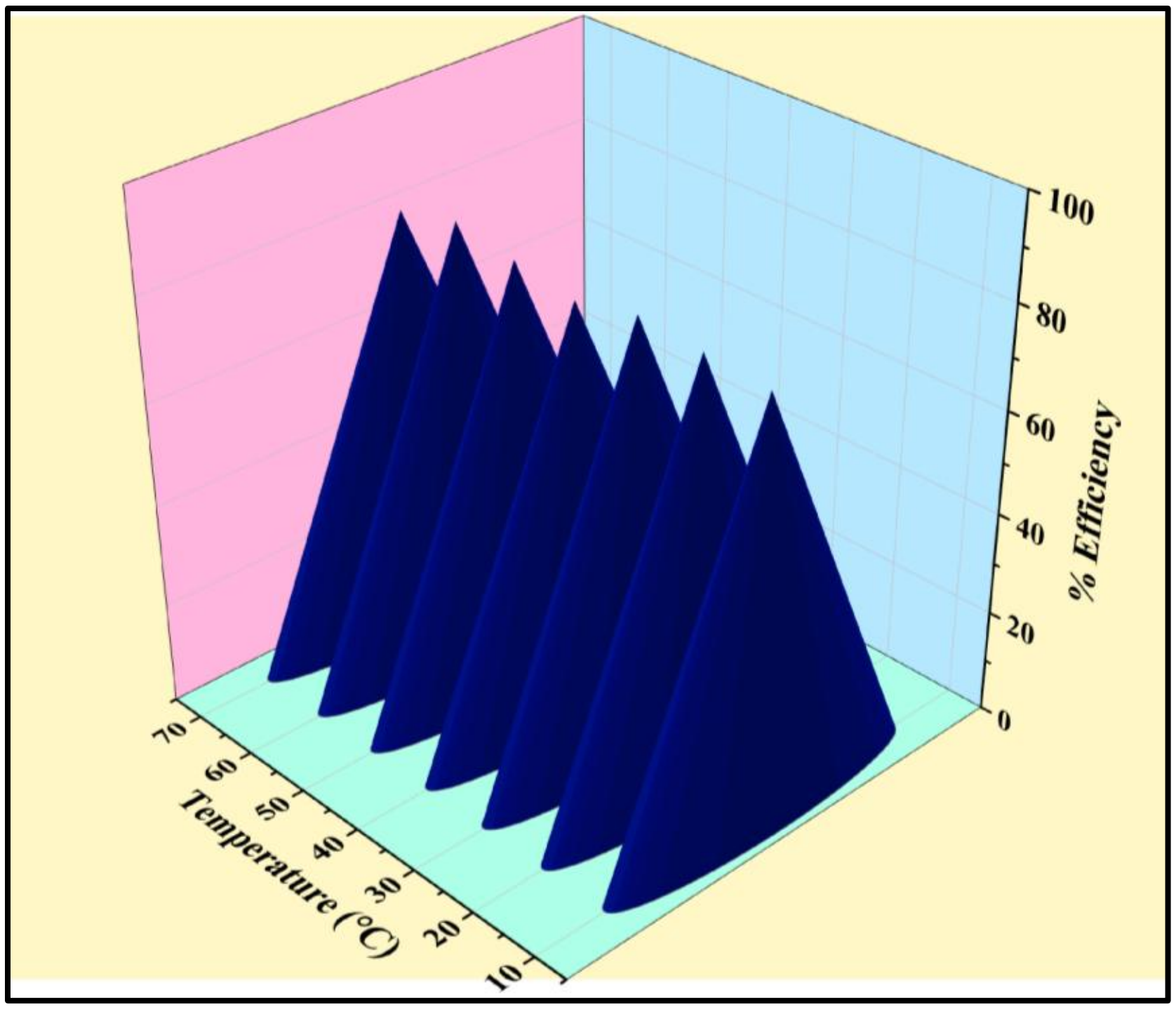

Figure S16: Interference study showing the effect of temperature (10-70 °C) on the fluorescence quenching efficiency of the **DPD@Fe^{2+}** system.

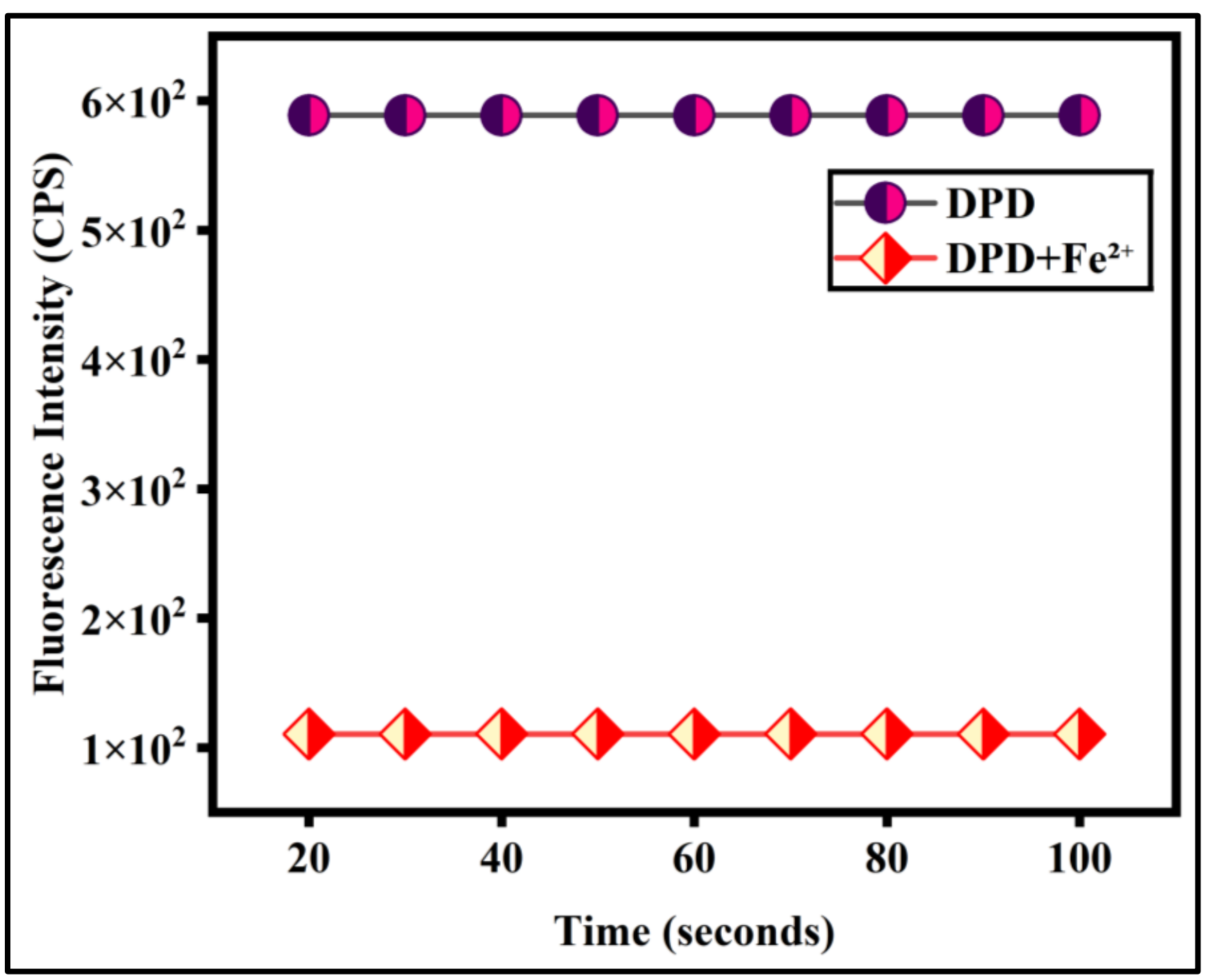

Figure S17: Photostability of **DPD** and the **DPD@Fe^{2+}** system under continuous irradiation.

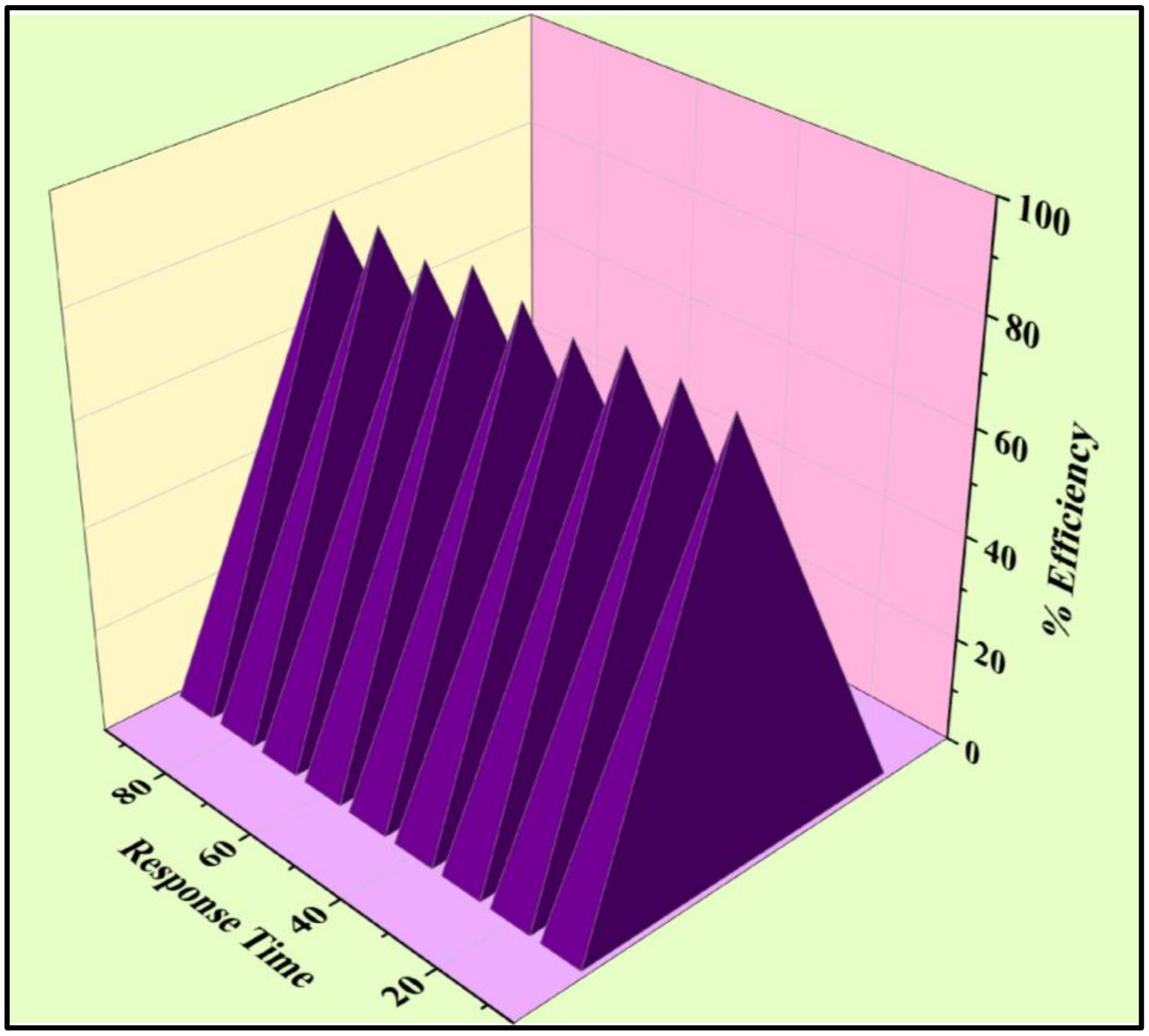

Figure S18: Effect of response time on the sensing efficiency of **DPD** toward Fe^{2+}.

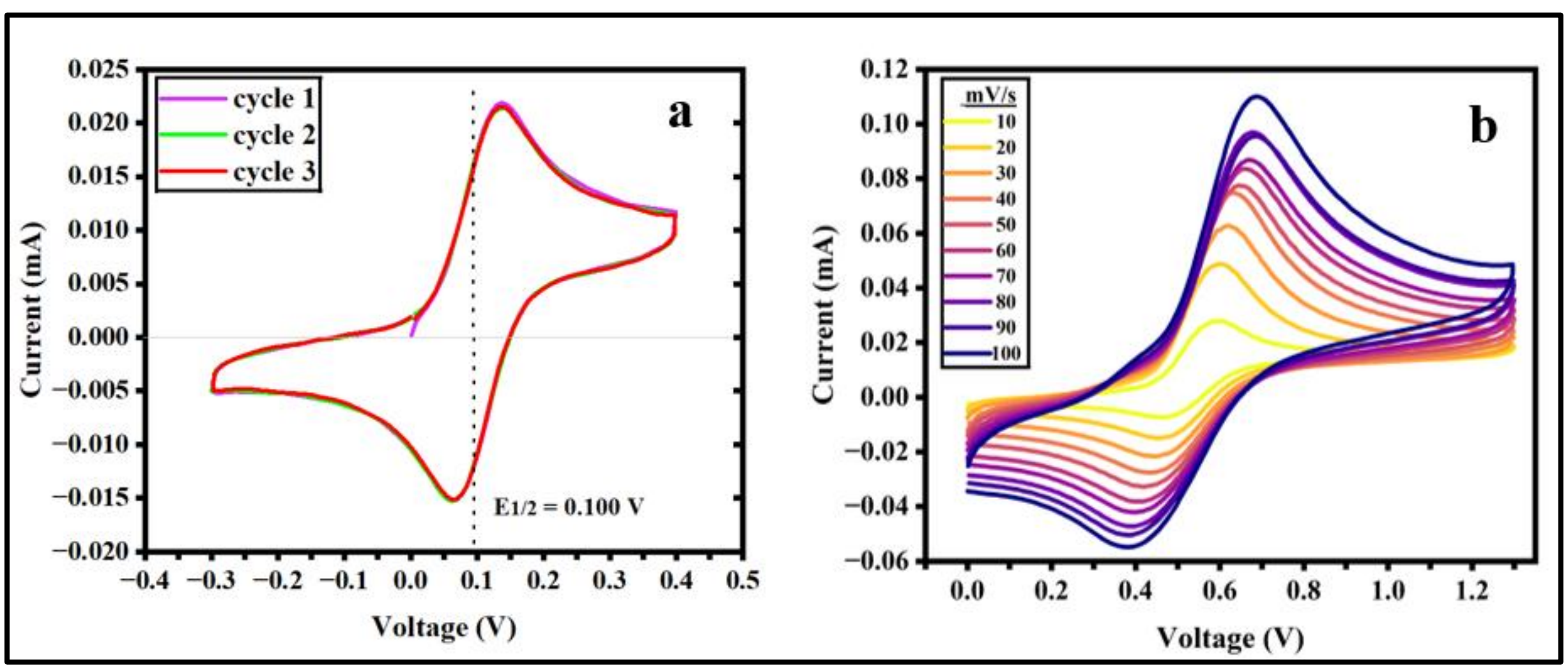

Figure S19: Cyclic voltammograms of ferrocene over three consecutive cycles, showing the Fc/Fc^+ half-wave potential (a). CV profiles of DPD recorded at scan rates of 10-100 mV s^{-1} (b).

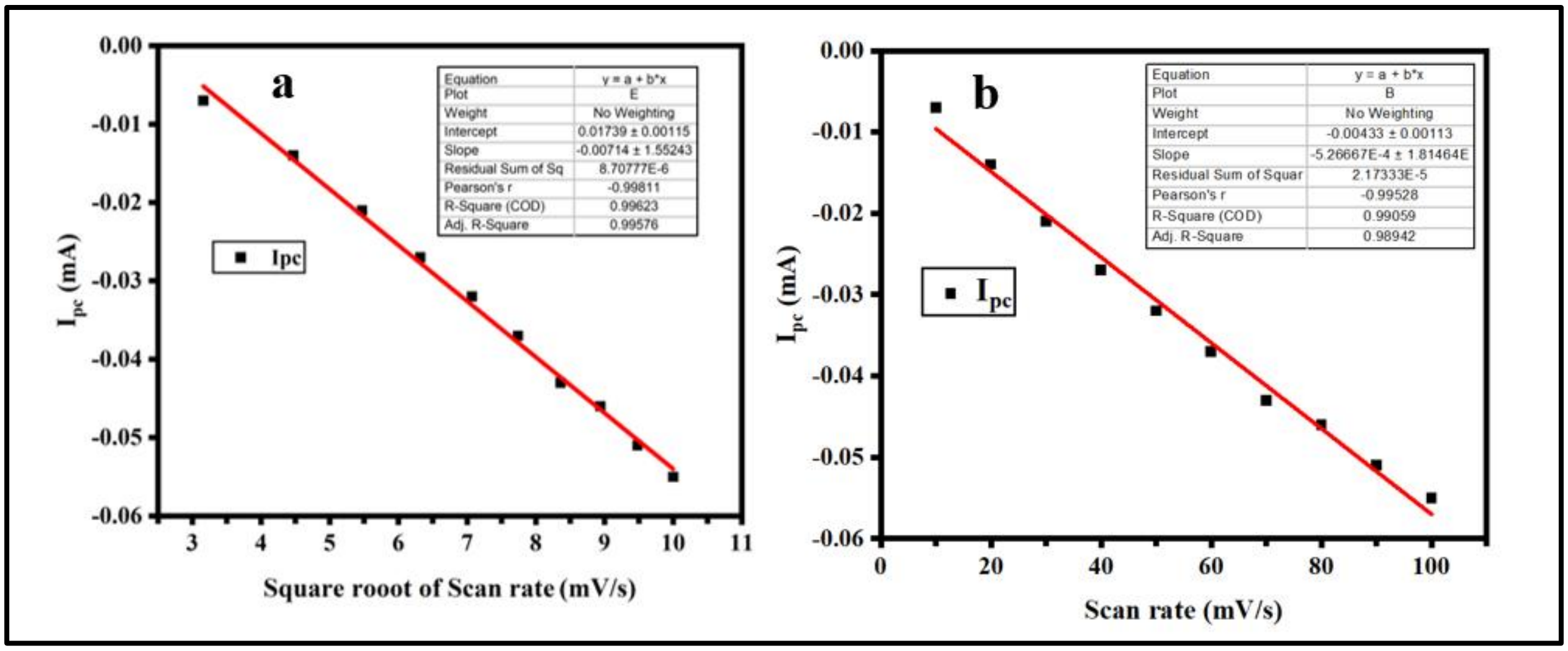

Figure S20: (a, b) Linear regression plots of cathodic peak current I_{pc} versus the square root of scan rate for DPD, showing the diffusion-controlled contribution to the electrochemical process ($R^2 = 0.99623$).

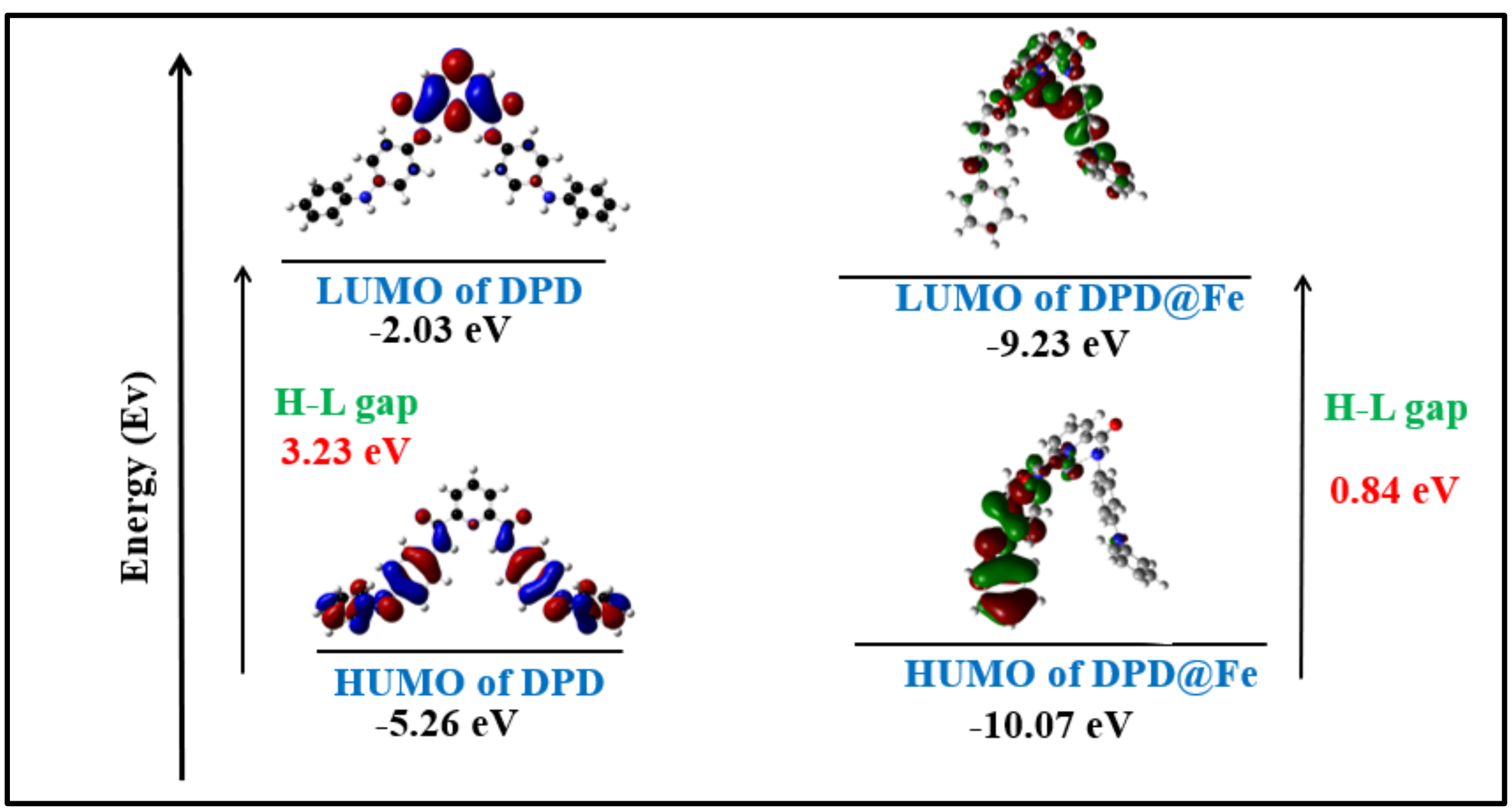

Figure S21: Frontier molecular orbital (HOMO-LUMO) energy levels and orbital distributions of free **DPD** and the **DPD@Fe²⁺** complex obtained from DFT calculations.

Table S2: Calculated vibrational frequencies (cm^{-1}) and corresponding infrared intensities of the optimized DPD and DPD-Fe^{2+} structures.

Vibrational Frequency and Infrared Intensity Data

Mode	Freq.	IR	Mode	Freq.	IR	Mode	Freq.	IR
1	-96.31	1476.50	63	723.92	13.90	125	1310.38	90.85
2	-3.62	0.34	64	727.85	16.15	126	1316.42	103.39
3	15.95	2.44	65	736.64	102.47	127	1328.52	92.02
4	22.02	0.70	66	754.24	421.53	128	1343.19	1491.24
5	25.00	7.54	67	763.21	56.19	129	1351.35	188.34
6	34.88	14.91	68	781.79	79.63	130	1368.87	359.14
7	39.64	18.58	69	782.80	47.25	131	1374.19	77.09
8	48.35	4.08	70	793.30	88.07	132	1376.83	348.45
9	52.83	59.15	71	795.76	132.28	133	1381.42	661.14
10	60.41	1.73	72	825.13	56.08	134	1384.25	191.22
11	70.28	8.13	73	830.00	8.29	135	1396.43	1427.18
12	78.77	15.60	74	840.10	56.93	136	1419.97	597.90
13	93.79	8.61	75	843.39	102.63	137	1424.62	191.59
14	106.85	100.67	76	850.36	26.35	138	1440.79	54.25
15	118.94	111.15	77	861.00	46.89	139	1454.91	38.73
16	141.25	61.14	78	866.64	36.74	140	1476.82	7.87
17	161.97	110.10	79	867.77	21.21	141	1491.67	6.28
18	173.54	35.45	80	875.07	14.91	142	1498.67	49.83
19	177.57	9.14	81	875.99	48.07	143	1506.96	24.90
20	185.49	63.31	82	883.36	9.43	144	1518.03	52.44
21	203.94	174.51	83	890.86	7.54	145	1519.70	51.20
22	224.72	13.11	84	947.16	44.91	146	1531.48	694.35
23	232.24	92.17	85	954.50	4.69	147	1536.71	67.27
24	253.72	13.11	86	966.65	265.59	148	1549.65	929.70
25	276.77	168.78	87	967.11	4.50	149	1560.53	42.92
26	291.53	62.85	88	978.43	16.68	150	1573.68	2354.62
27	299.98	249.11	89	985.09	219.24	151	1589.05	22.69
28	307.29	10.03	90	994.34	48.64	152	1611.95	168.55
29	334.30	8.51	91	1005.47	0.21	153	1621.22	978.32
30	343.23	781.82	92	1010.68	34.63	154	1631.99	64.97
31	347.11	54.59	93	1012.31	3.52	155	1638.35	23.20
32	367.53	38.84	94	1014.53	14.11	156	1639.99	402.01
33	374.58	46.10	95	1020.09	0.68	157	1641.58	339.64
34	400.04	345.84	96	1021.05	5.12	158	1653.47	219.59
35	413.49	6.87	97	1022.37	4.92	159	1668.47	338.95
36	416.60	17.81	98	1026.11	12.47	160	1783.69	295.35
37	419.69	9.59	99	1030.78	3.53	161	1872.56	615.61
38	421.93	1.54	100	1043.02	1.09	162	3177.85	8.07
39	428.82	13.15	101	1044.36	0.23	163	3179.85	14.05
40	438.81	129.72	102	1046.62	3.14	164	3201.16	0.91
41	464.43	2.91	103	1048.48	5.52	165	3205.87	2.02
42	484.41	82.23	104	1054.40	0.28	166	3210.25	3.68
43	494.67	47.23	105	1066.10	0.23	167	3210.45	4.35
44	505.85	89.97	106	1072.26	172.55	168	3217.09	12.96
45	515.41	216.49	107	1103.40	102.02	169	3217.34	6.44
46	521.84	48.66	108	1116.06	9.93	170	3225.72	0.92
47	531.70	26.54	109	1117.99	4.84	171	3225.77	1.06
48	578.87	6.99	110	1158.40	400.60	172	3228.00	3.88
49	595.12	9.71	111	1168.66	106.58	173	3233.57	1.70
50	600.84	79.02	112	1180.66	679.14	174	3234.00	16.69
51	611.91	27.84	113	1199.84	81.43	175	3235.56	6.98
52	625.48	2.87	114	1211.98	8.46	176	3240.02	446.71
53	628.40	2.87	115	1216.59	4.36	177	3248.39	25.55
54	631.32	6.68	116	1217.66	87.21	178	3251.00	9.85
55	635.45	33.10	117	1219.59	31.07	179	3257.48	15.67
56	658.42	86.12	118	1220.43	486.86	180	3260.47	5.35
57	662.93	22.00	119	1234.55	891.68	181	3262.77	2.16
58	667.70	120.18	120	1237.48	32.03	182	3265.61	307.68
59	690.60	55.67	121	1238.07	328.60	183	3287.70	275.32
60	702.60	139.35	122	1252.07	38.51	184	3498.80	70.05
61	708.49	51.92	123	1254.66	0.85	185	3567.28	163.98
62	714.21	67.57	124	1299.79	90.83	186	3598.00	142.93

Table S3: QTAIM topological parameters and interaction energies for the bond critical points (BCPs) in the DPD-Fe^{2+} complex.

BCPs	Probe-analyte	ρ(r) (a.u.)	∇²ρ(r) (a.u.)	G(r) (a.u.)	V(r) (a.u.)	H(r) (a.u.)	−V/G	Eint (kcal/mol)
87	N···Fe^{2+}	0.0851	0.3560	0.1110	-0.1330	-0.0220	1.198	-174.51
91	N···Fe^{2+}	0.1117	0.6564	0.1973	-0.2305	-0.0332	1.168	-302.44
98	Fe^{2+}···N	0.0827	0.4077	0.1220	-0.1422	-0.0201	1.165	-186.58
106	Fe^{2+}···C	0.0752	0.2431	0.0748	-0.0887	-0.0139	1.019	-1163.99

Complete Reference for Gaussian 09

Gaussian 09, Revision D.01,M. J. Frisch, G. W. Trucks, H. B. Schlegel, G. E. Scuseria, M. A. Robb, J. R. Cheeseman, G. Scalmani, V. Barone, B. Mennucci, G. A. Petersson, H. Nakatsuji, M. Caricato, X. Li, H. P. Hratchian, A. F. Izmaylov, J. Bloino, G. Zheng, J. L. Sonnenberg, M. Hada, M. Ehara, K. Toyota, R. Fukuda, J. Hasegawa, M. Ishida, T. Nakajima, Y. Honda, O. Kitao, H. Nakai, T. Vreven, J. A. Montgomery, Jr., J. E. Peralta, F. Ogliaro, M. Bearpark, J. J. Heyd, E. Brothers, K. N. Kudin, V. N. Staroverov, T. Keith, R. Kobayashi, J. Normand, K. Raghavachari, A. Rendell, J. C. Burant, S. S. Iyengar, J. Tomasi, M. Cossi, N. Rega, J. M. Millam, M. Klene, J. E. Knox, J. B. Cross, V. Bakken, C. Adamo, J. Jaramillo, R. Gomperts, R. E. Stratmann, O. Yazyev, A. J. Austin, R. Cammi, C. Pomelli, J. W. Ochterski, R. L. Martin, K. Morokuma, V. G. Zakrzewski, G. A. Voth, P. Salvador, J. J. Dannenberg, S. Dapprich, A. D. Daniels, O. Farkas, J. B. Foresman, J. V. Ortiz, J. Cioslowski, and D. J. Fox, Gaussian, Inc., Wallingford CT, 2013.

References

1. Revanna, B. N., Kamat, V., Swamynayaka, A., Harish, K. K., Venkatesha, K., Madegowda, M and Kollur, S. P. (2025). Chalcone-based turn-off chemosensor for selective and susceptible detection of Fe^{2+} ions: spectroscopic and DFT investigations. *Journal of Fluorescence*, *35*(3), 1781-1795.
2. Sasan, S., Chopra, T., Gupta, A., Tsering, D., Kapoor, K. K., & Parkesh, R. (2022). Fluorescence "turn-off" and colorimetric sensor for Fe^{2+}, Fe^{3+}, and Cu^{2+} ions based on a 2, 5,7-triarylimidazopyridine scaffold. *ACS Omega*, *7*(13), 11114.
3. Gul, P., Koseoglu, T.S., and Ekmekci, Z., An imidazole-functionalized boron-dipyrromethene derivative for spectrophotometric fluorescence detection of Fe^{2+} and electrochemical sensing of Cu^{2+}. ChemistrySelect, 2026. 11(5): p. e05412.
4. Asiwal, E. P., Shelar, D. S., Gujja, C. S., Manjare, S. T., & Pawar, S. D. (2022). A Ni-MOF based luminescent sensor for selective and rapid sensing of Fe (II) and Fe (III) ions. *New Journal of Chemistry*, *46*(26), 12679-12685.
5. Ömeroğlu, İ. and Şanko, V., A "turn-off" fluorescence sensor for Fe^{2+}, Fe^{3+}, and Cu^{2+} ions based on novel pyrene-functionalized chitosan. Turkish Journal of Analytical Chemistry, 2023. 5(1): p. 50–60.
6. Kumar, Y., Kaushik, R., Rani, S., Rafat, S., Shabir, J., Dev, K., & Kumar, L. S. (2021). Curcumin immobilized metal organic framework based fluorescent nanoprobe for selective sensing and bioimaging of Fe (II). *Materials Today Communications*, *28*, 102563.
7. Mitcharean, C., Kedsakon, K., Hasdin, O., Busayaporn, W., Chunhakowit, P., Songsrirote, K., & Prayongpan, P. (2026). Fluorescent poly (vinyl alcohol) composite films incorporating doped carbon dots for heavy metal detection in smart packaging applications. *ACS Omega*, *11*(16), 24188.
